# Betaine as a regulator of metabolism, epigenetics, and cellular osmoprotection: therapeutic implications in cardiometabolic and renal diseases

**DOI:** 10.3389/fphar.2026.1883688

**Published:** 2026-07-31

**Authors:** Sumet Kumar Singh, Poonam Yadav, Manish Bishnolia, Nirmal Manhar, Puneet Kumar, Umashanker Navik, Ralf Weiskirchen

**Affiliations:** 1 Department of Pharmacology, Central University of Punjab, Bathinda, India; 2 Institute of Molecular Pathobiochemistry, Experimental Gene Therapy and Clinical Chemistry (IFMPEGKC), RWTH Aachen University Hospital, Aachen, Germany

**Keywords:** betaine, epigenetics, health and diseases, methyl donor, osmoprotectant

## Abstract

Betaine, a glycine derivative found in spinach, beets, and whole grains, offers significant pharmacological and nutritional benefits. Researchers are focusing on betaine due to its therapeutic properties and ease of dietary incorporation. The zwitterion betaine is a derivative of N,N,N-trimethylglycine, which is produced during choline metabolism. However, endogenous synthesis of betaine may not always meet the health requirements, and dietary supplementation is needed. Betaine also acts as a methyl donor and an osmolyte and has proven beneficial in treating conditions such as hyper homocysteinemia, inflammation, cardiovascular disorders, and metabolic disorders. Pre-clinical and clinical studies demonstrate its therapeutic effectiveness, supported by safety reports confirming its role in enhancing human health. This review aims to summarize the biochemical, molecular, and therapeutic roles of betaine across multiple disease conditions, with a particular focus on its role in metabolic liver diseases (ALD/MASLD/MASH), cardiovascular dysfunction, diabetes, and renal disorders. Additionally, it highlights betaine’s emerging role as an epigenetic regulator and assesses its safety and toxicity profile. Betaine primarily acts as a methyl donor in the one-carbon metabolism pathway, reducing hyperhomocysteinemia and attenuating cardiovascular risk. In hepatic metabolism, betaine modulates lipid homeostasis by regulating key lipogenic transcription factors, contributing to its anti-steatotic and anti-inflammatory effects in MASLD/MASH. Its osmoprotective properties maintain cellular integrity during stress conditions. Furthermore, betaine regulates epigenetic modulations of genes linked to inflammation, fibrosis, and metabolic pathways. Betaine is generally regarded as non-toxic. However, its dose-dependent effects and long-term safety require further analysis, along with detailed evaluations of its multifaceted protective mechanism against disease conditions. Collectively, betaine represents a promising therapeutic candidate with significant translational potential for the treatment of metabolic, cardiovascular/cardiometabolic, and renal diseases.

## 1 Introduction

Betaine (also known as trimethylglycine) is a zwitterionic quaternary ammonium compound that is widely used in food processing due to its excellent water solubility. Over 50 different types of betaine have been identified (Székely and Máté, 2022). Physiologically, betaine is an amino-acid derivative that is considered a non-essential nutrient for the body (Gilbert-Barness et al., 2017). The human body produces betaine internally from free choline in the presence of the enzyme choline dehydrogenase. During metabolism, betaine is converted into dimethylglycine, sarcosine, and glycine by the enzymes betaine-homocysteine methyltransferase (BHMT), dimethylglycine dehydrogenase (DMGDH), and sarcosine dehydrogenase, respectively (Hoffmann et al., 2013). However, the endogenous production is insufficient to regulate physiological functions, and humans require additional external dietary sources (Wiedeman and Alejandra, 2017). Primary exogenous sources of betaine include spinach, beets, and whole grains such as wheat, barley, brown rice, quinoa, and oats (Dobrijević et al., 2023). Additionally, betaine can be chemically produced or extracted from sugar beets and processing byproducts. Due to its numerous benefits, natural betaine is preferred by the pharmaceutical, cosmetic, and healthcare industries over its synthetic alternative (Altinisik et al., 2023). Additionally, betaine has low toxicity, as a daily intake of 9 g–15 g is considered safe for human consumption.

Betaine offers various therapeutic and nutritional benefits. Numerous studies have explored its efficacy in pre-clinical and clinical models against various disorders. Betaine significantly improved gastrointestinal, renal, cardiac, and hepatic complications (Kaur et al., 2019). These cytoprotective actions were thought to be caused by betaine’s antioxidant, anti-inflammatory, and anti-apoptotic properties (Gao et al., 2017). Betaine donates a methyl group for BHMT-mediated remethylation of homocysteine to methionine. DNA and proteins use this methyl group for methylation (Dobrijević et al., 2023). Betaine is a well-known osmolyte that helps remove waste products through the urine and reduces osmotic stress to protect cells (Ross et al., 2014).

However, current dietary and lifestyle modifications have increased the burden of most diseases. These changes lead to physiological alterations in the body, thus initiating disease pathology by generating free radicals and increasing oxidative stress (Bodden et al., 2021). Medications taken to cure diseases also have secondary effects that can worsen the conditions (Bodden et al., 2021). Researchers are focusing more on natural compounds such as betaine because of their low toxicity, low cost, and better therapeutic properties. Moreover, it can be easily incorporated into human diet.

Altogether, this review aims to comprehensively examine betaine, with a specific focus on its metabolism and therapeutic properties in the treatment of various disorders. The review emphasizes betaine’s bioactive properties and health benefits, including its antioxidant, anti-inflammatory, and cytoprotective actions. Furthermore, it addresses betaine’s safety and efficacy through pre-clinical and clinical studies. Ultimately, the review underscores the potential of betaine as a cost-effective natural alternative to synthetic drugs, with fewer side effects.

## 2 Methodology

A comprehensive literature search was performed using the PubMed, Scopus, Web of Science, Google Scholar, and ClinicalTrials.gov databases to identify studies analyzing the biochemical, molecular, pharmacological, and therapeutic roles of betaine.

The search strategy included combinations of the following keywords: “betaine,” “trimethylglycine,” “one-carbon metabolism,” “methyl donor,” “homocysteine,” “epigenetics,” “DNA methylation,” “osmoprotection,” “cardiovascular disease,” “metabolic disorders,” “diabetes,” “metabolic dysfunction-associated steatotic liver disease/non-alcoholic fatty liver disease,” “metabolic dysfunction-associated steatohepatitis/non-alcoholic steatohepatitis,” “alcohol-associated liver disease,” “inflammation,” “oxidative stress,” “kidney disease,” “renal disease,” and “toxicity.”

The search included publications from June 1988 to November 2025. Original research articles, pre-clinical studies, clinical trials, mechanistic studies, case reports, and relevant review articles were considered for inclusion. Studies focusing on betaine’s metabolism, epigenetic regulation, osmoprotective functions, pharmacological mechanisms, therapeutic applications, and safety profile were prioritized. Duplicate records, conference abstracts, studies with insufficient methodological detail, and publications not directly relevant to the review’s objectives were excluded. Additionally, relevant publications were identified by manually searching the reference lists of selected articles.

Over 350 references were retrieved in total, of which 197 were selected for inclusion after critical evaluation to provide a comprehensive overview of the current understanding of betaine as a regulator of metabolism, epigenetics, and osmoprotection. Furthermore, the therapeutic implications of betaine in cardiometabolic, hepatic, and renal diseases, along with its relevant pre-clinical and clinical studies and safety profiles, have been incorporated.

## 3 Metabolism of betaine

**FIGURE 1:**
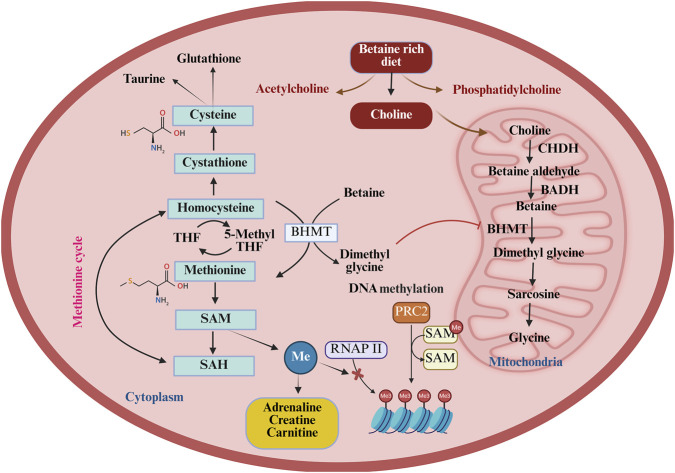
Metabolism of betaine. Dietary betaine can be obtained directly from food sources or generated endogenously from choline. Choline is oxidized by choline dehydrogenase (CHDH) to form betaine aldehyde, which is then converted into betaine with the help of betaine aldehyde dehydrogenase (BADH). Betaine acts as a methyl donor in one-carbon metabolism, participating in betaine-homocysteine methyltransferase (BHMT)-mediated remethylation of homocysteine to methionine. This process produces dimethylglycine (DMG) as a by-product. DMG is then metabolized to sarcosine and subsequently to glycine. Alternatively, homocysteine can be remethylated to methionine via the folate-dependent pathway involving 5-methyltetrahydrofolate (5-methyl THF) and tetrahydrofolate (THF). Methionine is then converted into S-adenosylmethionine (SAM), which is the universal methyl donor for numerous cellular methylation reactions. After a methyl group is transferred, SAM is converted to S-adenosylhomocysteine (SAH), which is then hydrolyzed to homocysteine, thus completing the methionine cycle. Alternatively, homocysteine can enter the trans-sulfuration pathway to produce cystathionine, cysteine, glutathione, and taurine. Methyl groups derived from SAM are utilized in DNA methylation, histone modifications, and the biosynthesis of methylated compounds such as creatine, carnitine, and adrenaline. 5-Methyl THF, 5-methyltetrahydrofolate; BADH, betaine aldehyde dehydrogenase; BHMT, betaine-homocysteine methyltransferase; CHDH, choline dehydrogenase; DMG, dimethylglycine; Me, methyl group; PRC2, polycomb repressive complex 2; RNAP II, RNA polymerase II; SAH, S-adenosylhomocysteine; SAM, S-adenosylmethionine; THF, tetrahydrofolate.

Chemically, betaine is a trimethylglycine that is converted into dimethylglycine (DMG) and, finally, into sarcosine in the mitochondria of liver and kidney cells (Day and Kempson, 2016). Betaine’s primary function is to regulate blood homocysteine levels. Specifically, since homocysteine accumulation is detrimental to cells, betaine converts it into other beneficial compounds to maintain stable amino acid levels (Figueroa-Soto and Valenzuela-Soto, 2018). Betaine’s essential physiological roles include providing a methyl group for homocysteine remethylation, preventing protein denaturation by acting as a chemical chaperone, and regulating organic osmolytes within cells to control the cell volume (Alvarenga et al., 2022). After consuming betaine-rich foods, it is absorbed in the duodenum of the small intestine and distributed within 1 h–2 h after digestion. It is typically found in human serum at levels ranging from 20 to 70 μmol/L, with an elimination half-life of 14.38 h (Dobrijević et al., 2023). Choline biotransformation contributes to the synthesis of endogenous betaine. In mitochondria, choline is first oxidized by choline dehydrogenase (CHDH) to form betaine aldehyde. This is then converted into betaine by betaine aldehyde dehydrogenase (BADH/ALDH9A1) (Day and Kempson, 2016). Betaine then serves as a methyl donor in the BHMT-mediated remethylation of homocysteine to methionine, producing DMG as a by-product. DMG is then metabolized further to sarcosine, which is subsequently converted to glycine (Dobrijević et al., 2023). Homocysteine can also be remethylated to methionine via the folate-dependent pathway. In this process, 5-methyltetrahydrofolate (5-MTHF) donates a methyl group to homocysteine, leading to the formation of tetrahydrofolate (THF) and methionine (Dobrijević et al., 2023). Methionine is then converted to S-adenosylmethionine (SAM), which is subsequently converted to S-adenosylhomocysteine (SAH), followed by demethylation. One molecule of SAH hydrolyzes to form one molecule of homocysteine and adenosine, completing the methionine cycle. The methyl group is further used to synthesize amino acids and other compounds, such as adrenaline, creatine, and carnitine (Zhao et al., 2018) (Figure 1).

Due to this, betaine serves as a valuable source of methyl, and the methylation process is essential for the synthesis of protein and DNA. It is primarily excreted through the skin and urine, especially in individuals with diabetes and kidney disease. Betaine helps detoxify homocysteine by converting it to methionine. Elevated blood homocysteine levels can indicate various conditions, including cardiovascular, renal, cerebral, vascular, cognitive, and neurological disorders (Smith and Refsum, 2021).

## 4 Therapeutic effects

The significant impact of betaine on osmosis, diabetes mellitus, cardiovascular disorders, liver function, inflammation, hyper homocysteinemia, and other diseases has been well-demonstrated from cellular to molecular perspectives. In the following section, we will discuss this in further detail.

### 4.1 Betaine as an osmolyte

**FIGURE 2:**
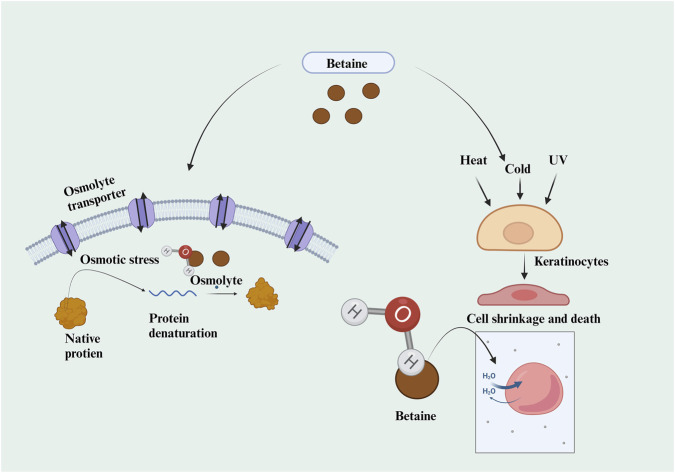
Pathogenesis of water imbalance. Environmental stressors such as heat, cold, and ultraviolet (UV) radiation can induce hyperosmotic stress in keratinocytes, leading to cellular dehydration and shrinkage, along with impaired cellular function. In response, cells upregulate the expression of osmolyte transporters in order to take up and accumulate compatible osmolytes, such as betaine. As an organic osmolyte, betaine helps maintain intracellular osmotic balance, promotes water retention, and protects cells against damage induced by osmotic stress. It also helps to maintain the native conformation of proteins and reduce denaturation caused by osmotic stress, thereby maintaining protein structure and cellular integrity. Thus, betaine contributes to maintaining cell volume, protein homeostasis, and overall cellular survival under stressful environmental conditions.

Osmolytes play a critical role in regulating the rate and size of cell growth. Cells accumulate suitable organic osmolytes such as betaine, sorbitol, taurine, and glycerophosphocholine in response to the stress of long-term hyperosmolality, maintaining normal cell volume and electrolyte contents (Alhuthali et al., 2021). Osmolytes are classified into the following groups: free amino acids and their derivatives, methylamines, saccharides, polyols, and ectoines. Betaine falls under the methylamines group (Ilyas et al., 2022). The unique chemistry of betaine makes it a specific osmolyte and chemical chaperone. By preventing direct contact with unfolded proteins, betaine contributes to the entropic mechanism of osmolyte-mediated protein stabilization (Figure 2). Interestingly, to maintain water homeostasis within the cell, betaine binds to hydrogen bonds in water molecules and carries them back to the cell (Stasiulewicz et al., 2022). Additionally, salt and water, due to their higher concentration, bind to proteins and destabilize them. However, betaine does not bind to proteins because the protein backbone is osmophobic in nature (Yancey, 2005). Mechanistically, betaine contributes to intracellular water and osmotic homeostasis by accumulating as a compatible osmolyte that balances osmotic gradients, influences the hydration shells of biomolecules, and is preferentially excluded from protein surfaces; these effects favor native protein conformations and reduce osmotic-stress-induced protein denaturation (Yancey, 2005; Stasiulewicz et al., 2022). As it does not harm cells even at high concentrations, betaine is an example of a non-perturbing osmolyte. In the renal medulla, high concentrations of NaCl and urea create a strongly hyperosmotic environment that can destabilize cellular proteins; renal medullary cells therefore accumulate compatible osmolytes, including betaine, which counterbalance osmotic stress, preserve cell volume, and support protein stability through chemical-chaperone-like effects (Neuhofer and Beck, 2005; Neuhofer et al., 2005).

### 4.2 Role of betaine in hyper homocysteinemia and homocystinuria

Homocysteine is a non-essential amino acid produced in the body as a result of the metabolism of methionine. Furthermore, homocysteine is a necessary intermediate in synthesizing two amino acids, cysteine and methionine. The normal range of homocysteine is 5 μmol/L–15 μmol/L. When the levels exceed 15 μmol/L, it is called hyperhomocysteinemia (Veeranki et al., 2016). Elevated levels of homocysteine in the blood can lead to various cardiovascular conditions, such as coronary artery disease, myocardial infarction (MI), and stroke, along with cognitive impairment (Yuan et al., 2021). The blood homocysteine level can be increased by defects in the enzyme methylene tetrahydrofolate reductase (MTHFR) that catalyzes the breakdown of methionine and due to deficiencies in folate, vitamin B6, and B12 (Park and Chang, 2014). Generally, methionine from dietary intake gets converted to SAM in a reaction catalyzed by methionine-adenosyl-transferase. SAM is the key methyl donor in various cellular methylation reactions. By donating a methyl group to various substrates, it forms SAH, which is converted to homocysteine by S-adenosylhomocysteine hydrolase (Dai et al., 2022).

Meanwhile, it has been reported that betaine lowers the overall homocysteine concentration by transmethylation of homocysteine into methionine in the BHMT reaction and protects rats from ethanol-induced oxidative stress as a methyl donor (Alirezaei et al., 2015). In this transmethylation event, betaine donates a methyl group to homocysteine, synthesizing methionine. In the folate-dependent remethylation pathway, 5-methyltetrahydrofolate (5-MTHF) serves as the methyl donor, while vitamin B12-dependent methionine synthase catalyzes the transfer of this methyl group to homocysteine, thereby forming methionine (Yeroshkina et al., 2023). A study in South China revealed that daily supplementation with low-dose vitamins B and betaine (400 μg folic acid, 8 mg vitamin B6, 6.4 µg vitamin B12, and 1 g betaine) significantly reduced the concentrations of homocysteine in plasma in Chinese adults with hyperhomocysteinemia (Lu et al., 2023).

Homocystinuria is a rare genetic disorder where the body cannot fully break down methionine, leading to the accumulation of methionine and homocysteine. This condition is characterized by osteoporosis, skeletal deformities, and blood-clotting issues, among others. It is caused by a deficiency in the enzyme CBS, which is involved in the metabolism of homocysteine (Truitt et al., 2021). Treatment aims to lower the homocysteine levels in patients with homocystinuria. Betaine is recommended for those who do not respond to vitamin B6 supplementation (Lu et al., 2023). Vitamin B6 helps the enzyme function, while betaine acts as a methyl donor, converting homocysteine to methionine (Zuhra et al., 2020). Betaine, along with vitamin B12 supplements, plays a crucial role in remethylating homocysteine back to methionine. Vitamin B12 is a cofactor for methionine synthase. These reactions are important for maintaining methionine levels and preventing cardiovascular disease (CVD) and skeletal abnormalities.

One study analyzed the safety of betaine anhydrous in treating patients with inherited homocystinuria. The study included 125 patients aged 18 or older with inherited homocystinuria who were treated with betaine anhydrous in combination with other strategies. The daily dose of betaine was 6 g/day for CBS-deficient patients and 9 g/day for those lacking methylenetetrahydrofolate reductase. Patients receiving betaine experienced an overall 29% decrease in plasma homocysteine levels. This study provided evidence of betaine anhydrous’ clinical safety and efficacy in the management of homocystinuria (Valayannopoulos et al., 2019). Overall, betaine is important for the management of hyperhomocysteinemia, homocystinuria, and their associated complications.

### 4.3 Role of betaine in inflammation

Severe or chronic inflammation can contribute to the pathophysiology of various diseases, as elevated ROS levels are likely to trigger pathological processes (Freitas et al., 2016). Oxidative metabolism in the mitochondria produces ROS, which can impair cell function and promote inflammation (Jomova et al., 2023). Betaine has been reported to suppress inflammatory mediators and signaling molecules, including cluster of differentiation 14 (CD14), tumor necrosis factor-α (TNF-α), cyclooxygenase-2 (COX-2), lipopolysaccharide (LPS)-induced TNF-α, toll-like receptor 2 (TLR2), TLR4, programmed cell death protein 4 (PDCD4), interleukin-1β (IL-1β), and nitric oxide synthase 2 (NOS2), and to prevent alcohol-induced hepatic cysteine and glutathione depletion (Medici et al., 2014).

One of the important targets for treating inflammation is the transcription factor NF-κB. Betaine has the ability to inhibit NF-κB activity and its downstream targets, such as TNF-α, IL-1β, and IL-23 (Hagar et al., 2019). Furthermore, betaine has been reported to suppress NF-κB-inducing kinase (NIK)/IκB kinase (IKK) and mitogen-activated protein kinase (MAPK) signaling, which are implicated in inflammatory activation and pro-inflammatory cytokine release (Kucharzewska et al., 2019). Betaine suppresses upstream signaling molecules that trigger NF-κB activation and maintains thiol levels, mainly glutathione, to prevent ROS generation and NF-κB activity (Go et al., 2007). Research has demonstrated that betaine administration suppresses NF-κB activation triggered by LPS and lowers the high-mobility group box 1 protein’s expression levels, which govern the activation of TLR-4 to reduce inflammation (Zhang et al., 2013). Another study found the role of betaine in inhibiting the activation of NLR family pyrin domain-containing 3 (NLRP3) inflammasome. The NLRP3 inflammasome is a complex protein that regulates inflammation. A study has demonstrated that betaine can increase the expression of heme oxygenase-1 (HO-1), which inhibits the NLRP3 inflammasome and reduces inflammation (Khodayar et al., 2020). Another study has demonstrated that betaine treatment can effectively decrease pro-inflammatory cytokines and NLRP3 inflammasome-related proteins in a dose-independent manner (Ge et al., 2016). This process is linked to the suppression of thioredoxin-interacting protein (TXNIP) by forkhead box O1 (FOXO-1), which increases ROS generation and NLRP3 inflammasome formation (Kim et al., 2017). Furthermore, betaine suppresses apoptosis by downregulating the caspase family proteins and activating transcription factor 3 (ATF-3) and apoptosis-related molecules such as caspase-3/7, caspase-8, and caspase-9 (Garrett et al., 2013).

Overall, betaine has great potential to alleviate diseases involving inflammation by regulating the levels of NLRP3, cytokines, NF-κB, oxidative stress, and apoptosis. However, betaine’s role in pathophysiology is not limited only to its anti-inflammatory properties. Further studies should focus more on the core mechanisms through which betaine mitigates inflammation.

### 4.4 Betaine’s role in cardiovascular diseases

According to the World Health Organization and the American Heart Association, CVDs encompass a range of disorders related to the heart and blood vessels and are the leading cause of death worldwide (World Health Organization, a; American Heart Association, 2026a).

#### 4.4.1 Betaine’s role in atherosclerosis

Atherosclerosis, a common form of CVD, is marked by inflammation, plaque accumulation, and lipid deposition in the arteries. This can cause blood flow to be blocked, either partially or completely, leading to serious problems such as stroke, coronary artery disease, angina, and myocardial infarction (American Heart Association, 2026b). Major risk factors for atherosclerosis include smoking, alcohol consumption, a Western diet, and lack of exercise (Wu et al., 2023). Over the past century, there has been significant understanding of the molecular pathology of atherosclerosis. Initially, atherosclerosis was defined by the buildup of cholesterol and thrombotic debris in the walls of arteries (Konstantinov et al., 2006). However, it is now recognized that the development of primordial plaques is chiefly influenced by inflammation, the proliferation of vascular smooth muscle cells (VSMCs), and the accumulation of lipoproteins, especially low-density lipoprotein (LDL) (Mannarino and Pirro, 2008). When macrophages take in these lipoproteins, they form lipid-laden foam cells, which were previously thought to be a sign of atheromata (Wojtasińska et al., 2023).

When inflammatory cytokines such as NLRP3 and NF-κB are activated, adhesion molecules such as vascular cell adhesion molecule-1 (VCAM-1), intercellular adhesion molecule-1 (ICAM-1), and monocyte chemoattractant protein-1 (MCP-1) are overexpressed (Libby, 2012; Dai et al., 2022). These events cause monocytes to adhere to the lining of an endothelium and promote VSMC proliferation, leading to the development and progression of atherosclerosis (Dai et al., 2022). For the past 6 decades, researchers have consistently found that hyperhomocysteinemia is a distinct risk indicator for CVD, especially atherosclerosis (Lentz, 2001). This condition, characterized by elevated levels of homocysteine in the blood, has been the focus of numerous studies aiming to mitigate atherosclerosis through homocysteine-lowering interventions (Yuan et al., 2023). Betaine has emerged as a promising approach against vascular inflammation caused by elevated homocysteine levels, thereby attenuating the progression of atherosclerosis (Ashtary-Larky et al., 2022a; Ashtary-Larky et al., 2022b). A study by Dai et al. found that in apolipoprotein E (ApoE)-deficient mice with SAHH deficiency, there was an accumulation of SAH and a decrease in the SAM/SAH ratio. This imbalance led to increased inflammation, proliferation, and migration of VSMCs, along with upregulation of adhesion molecules VCAM-1 and ICAM-1 and elevated levels of proinflammatory cytokines. Interestingly, when these mice were treated with betaine (4 g/100 g diet for 8 weeks), the pathological changes were reversed. The study noted a significant increase in the levels of BHMT, the enzyme responsible for converting homocysteine to methionine, indicating that betaine effectively counteracts the inflammatory and proliferative effects associated with hyperhomocysteinemia (Dai et al., 2022). Lv et al. (2009) studied the effects of betaine supplementation on atherosclerotic lesions in ApoE-deficient mice. The study demonstrated that ApoE deficiency in mice led to hypercholesterolemia and aortic inflammation, mediated by TNF-α, which contributed to the development of atherosclerotic lesions. Remarkably, the administration of betaine at various doses (0, 1, 2, or 4 g/100 g diet) significantly reduced aortic inflammation and TNF-α expression in a dose-dependent manner. These findings, along with the later study by Dai et al. (2022), underscore the potential of betaine as a therapeutic agent in managing atherosclerosis. By decreasing inflammation and the expression of pro-inflammatory cytokines, betaine shows promise in mitigating the progression of atherosclerotic disease in ApoE-deficient models (Lv et al., 2009).

In another interesting study, researchers examined the effects of either partially or entirely disrupting the *MTHFR* gene, which is essential for converting homocysteine to methionine. The disruption of this gene caused hyperhomocysteinemia, which led to inflammation of blood vessels, oxidative stress, and increased lipogenesis, causing the formation of atherosclerotic lesions (Schwahn et al., 2007). To explore potential interventions, the study examined the effects of betaine supplementation. For a short-term assessment, betaine was administered at 0.3% in drinking water (approximately 450 mg/kg body weight/day) for 2 weeks. For a long-term evaluation, betaine was included in the diet at 25 mmol/kg (approximately 300 mg/kg body weight) for 1 year. The results were promising, showing that betaine significantly reduced lipid accumulation in the aortic wall. Additionally, betaine increased the levels of apolipoprotein A-I (ApoA-I), a major protein component of high-density lipoprotein (HDL) known for its atheroprotective properties (Schwahn et al., 2007).

Moreover, Küskü-Kiraz et al. (2018) conducted a comprehensive study on the effects of betaine in a cholesterol–methionine (CHOL–Met) diet-induced atherosclerotic guinea pig model. The researchers noted that the CHOL–Met diet markedly increased the serum cholesterol and homocysteine concentrations. This diet also significantly reduced the production of endothelium-derived nitric oxide (NO) and increased the levels of asymmetric dimethylarginine (ADMA), which inhibits NO synthase from functioning. These changes damage endothelial cells and worsen atherosclerotic lesions. Notably, a 2% w/w dose of betaine treatment was able to counteract these adverse effects by restoring normal lipid profiles and NO levels and reducing oxidative stress. Furthermore, it alleviated aortic necroinflammation and fibrosis, which are critical indicators of atherosclerotic lesions.

A cohort study by Liu et al. (2023) confirmed the beneficial effect of betaine in the prevention of coronary artery disease. The study analyzed the correlation between dietary betaine intake and cardiovascular mortality in individuals diagnosed with coronary artery disease. After following 1,292 patients for an average of 9 years, the study found that consuming more dietary betaine was associated with a lower risk of mortality from heart disease.

**FIGURE 3:**
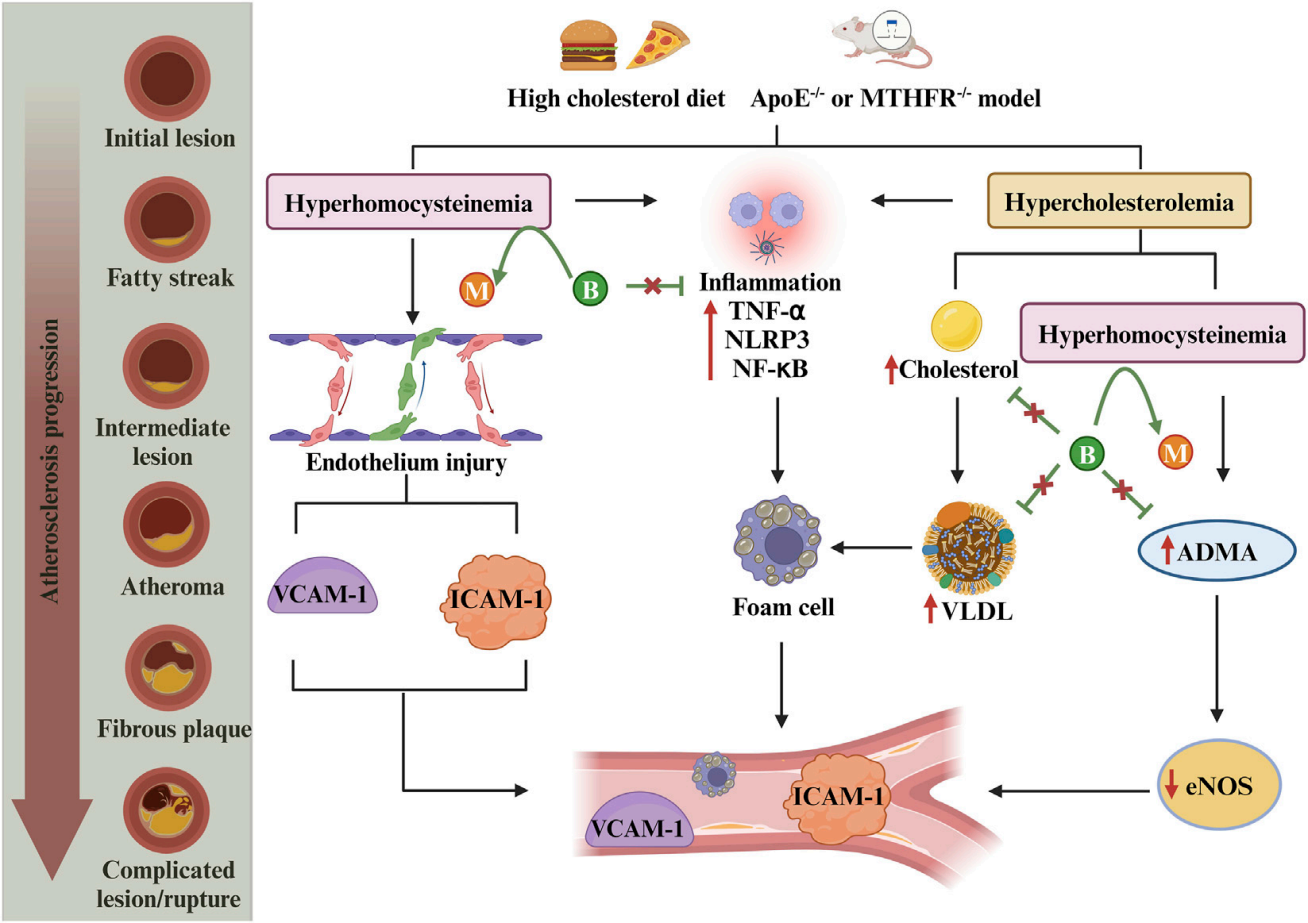
Schematic illustration of the progression of atherosclerosis and the role of hyperhomocysteinemia and hypercholesterolemia in endothelial injury and plaque formation. Atherosclerosis progresses from endothelial injury and the formation of fatty streaks to advanced fibrous plaques and plaque rupture. Hyperhomocysteinemia contributes to endothelial dysfunction by inducing vascular injury and upregulating adhesion molecules such as VCAM-1 and ICAM-1, which mediate leukocyte adhesion and vascular inflammation. Homocysteine also activates pro-inflammatory signaling pathways, including NF-κB, the NLRP3 inflammasome and TNF-α, thereby promoting foam cell formation and atherogenesis. Hypercholesterolemia promotes cholesterol accumulation and increases VLDL levels, accelerating the formation of foamy cells. Increased plasma concentrations of ADMA (an endogenous inhibitor of eNOS) further impair endothelial function and vascular homeostasis. Betaine (B) may attenuate the progression of atherosclerosis by reducing hyperhomocysteinemia, suppressing inflammatory signaling, improving endothelial function, and limiting the development of foam cells and atherosclerotic lesions. ADMA, asymmetric dimethylarginine; ApoE, apolipoprotein E; B, betaine; eNOS, endothelial nitric oxide synthase; ICAM-1, intercellular adhesion molecule-1; M, methionine; MTHFR, methylenetetrahydrofolate reductase; NF-κB, nuclear factor kappa B; NLRP3, NOD-like receptor pyrin domain-containing 3; TNF-α, tumor necrosis factor-α; VCAM-1, vascular cell adhesion molecule-1; VLDL, very low-density lipoprotein.

Betaine has significant potential in preventing the progression of atherosclerosis by suppressing inflammation, decreasing homocysteine levels, and improving lipid profiles. The evidence from multiple studies highlights betaine’s potential as a therapeutic agent in the management of atherosclerotic disease (Figure 3). Nevertheless, further research is necessary to fully elucidate the mechanism behind its protective effect.

#### 4.4.2 Betaine’s role in myocardial infarction

MI is clinically characterized by the necrosis of myocardial cells due to prolonged ischemia (Frangogiannis, 2015). The European Society of Cardiology (ESC), American College of Cardiology (ACC), American Heart Association (AHA), and World Health Federation (WHF) have established a fourth universal definition, stating: “MI denotes the presence of acute myocardial injury detected by abnormal cardiac biomarkers in the setting of evidence of acute myocardial ischemia” (Thygesen et al., 2018). Reports indicate that within 10 min–15 min of ischemia onset, the initial structural alterations in myocardial cells appear, probably due to reduced glycogen levels, which lead to relaxed myofibrils and sarcolemma injury. Coronary obstruction impairs energy metabolism, which leads to mitochondrial dysfunction and ultimately triggers the initiation of the apoptotic cascade (Thygesen et al., 2018; Smit et al., 2020).

In a series of studies, Ganesan et al. analyzed the protective effects of betaine on the isoprenaline-induced MI model using Wistar rats (Ganesan et al., 2007; Ganesan et al., 2010; Ganesan and Anandan, 2009; Ganesan et al., 2009). Pre-treating the rats with betaine (250 mg/kg per day) for 30 days prior to inducing MI showed significant attenuation of mitochondrial metabolic dysfunction and enhanced antioxidant levels in the myocardium. Betaine restored the ATP levels by modulating tricarboxylic acid (TCA) cycle enzymes, such as isocitrate dehydrogenase, succinate dehydrogenase, α-ketoglutarate dehydrogenase, and malate dehydrogenase. Furthermore, it modulated the levels of NADH-dehydrogenase and cytochrome c oxidase enzymes. Betaine also enhanced endogenous antioxidant defenses, reduced lipid peroxidation, and mitigated oxidative stress-induced myocardial damage (Ganesan et al., 2007; Ganesan et al., 2010). Furthermore, betaine stabilized lysosomal membranes by reducing the activities of lysosomal hydrolytic enzymes, thereby alleviating myocardial necrosis (Ganesan and Anandan, 2009). In addition, betaine preserved membrane-bound ATPase activity and maintained calcium, sodium, and potassium homeostasis by reducing calcium overload and membrane lipid peroxidation, thereby protecting against myocardial injury (Ganesan et al., 2009).

In addition to the anti-apoptotic, anti-inflammatory, and homeostasis of ATPase activity and minerals, betaine also exerts a cardioprotective effect through modulating signal transducer and activator of transcription 3 (STAT3). A study by Zheng et al. (2015) examined how betaine protects the heart in male Sprague-Dawley rats with isoproterenol-induced acute myocardial ischemia. The study focused on the modulation of the STAT3 and apoptotic pathways. Oral administration of betaine (200 and 400 mg/kg) for 40 days significantly reduced the cardiac injury biomarker cardiac Troponin I (cTnI), left ventricular hypertrophy, and histopathological alterations. The study demonstrated that betaine protects cardiac function by lowering the Bax/Bcl-2 ratio and altering the expression of the STAT3 protein. Furthermore, in 2017, Ghodratizadeh et al. (2017) administered isoproterenol to induce MI in albino rats. Rats that were treated with 250 mg/kg of betaine through oral gavage showed a significant decrease in homocysteine and cTnI levels.

Overall, betaine exhibits significant cardioprotective effects in MI by reducing oxidative stress, stabilizing mitochondrial function, and preserving membrane integrity. However, future studies must focus on determining the underlying molecular mechanisms behind its cardioprotection.

#### 4.4.3 Betaine’s role in hypertension

The global burden of hypertension has been increasing steadily over the years. According to a report in *The Lancet*, the number of adults with hypertension increased from 594 million in 1975 to 1.13 billion in 2015 (NCD Risk Factor Collaboration, 2017). However, a recent report by the WHO indicates that approximately 1.4 billion adults aged 30–79 years now suffer from hypertension (World Health Organization, b). Despite the numerous classes of anti-hypertensive drugs available, researchers have explored the effect of betaine in pre-clinical models of hypertension. In 2018, Yang et al. used monocrotaline to induce pulmonary arterial hypertension (PAH) in male Sprague-Dawley rats. PAH, a type of hypertension, is characterized by vascular remodeling, perivascular inflammation, smooth muscle cell proliferation, thrombosis, and vasoconstriction. NF-κB and TNF-α are key inflammatory pathways that play an important role in the pathogenesis of PAH (Wang Q. et al., 2013; Tang et al., 2021; Thenappan et al., 2018). Yang et al. (2018) demonstrated that betaine treatment (100, 200, and 400 mg/kg/day) over 21 days significantly reduced pulmonary arterial pressure and right ventricular hypertrophy. Furthermore, betaine effectively lowered vascular remodeling by decreasing the expression of inflammatory cytokines such as NF-κB, TNF-α, and IL-1β.

One of the major complications in PAH patients is that they often experience clinical worsening after an initial period of improvement with oral medications (Chakinala, 2009). In 2023, Che et al. conducted a study to assess the therapeutic efficacy of betaine based on the pharmacokinetic and pharmacodynamic factors and delivered it via various routes: intragastric administration, nebulized inhalation, and intravenous injection. The research indicated that nebulized inhalation of betaine produced the longest half-life and displayed significant biodistribution, particularly the notable accumulation of betaine in the lungs (Che et al., 2024). These results indicate that inhaled betaine is more effective in treating PAH-induced right ventricular failure due to the higher accumulation of betaine in the lungs, where it directly exerts its therapeutic effect.

Changes in the shape of the intimal, medial, and adventitial layers of blood vessels are characteristics of pulmonary vascular remodeling. This process includes inflammation, proliferation of smooth muscle cells, and fibrosis. Ultimately, these mechanisms trigger apoptotic pathways in cardiomyocytes, leading to RVF through the activation of the Ras homolog family member A/Rho-associated protein kinase (RhoA/ROCK) signaling pathway (Leopold and Maron, 2016; Tuder, 2017; Lv et al., 2021). In 2021, Lv et al. analyzed the potential therapeutic effects of betaine on RVF through the modulation of the Rho/ROCK signaling pathway in pulmonary hypertensive Sprague-Dawley rats. Administration of betaine at the doses of 100, 200, and 400 mg/kg/day significantly decreased the levels of brain natriuretic peptide (BNP), cTnI, and apoptosis markers, indicating its cardioprotective properties. Betaine also downregulated the expression of RhoA/ROCK and the associated apoptotic factors in pulmonary hypertensive rats (Lv et al., 2021).

Long-term elevation of arterial pressure leads to structural changes in the myocardium, leading to left ventricular hypertrophy. These changes create an imbalance between oxygen demand and supply, causing myocardial ischemia, infarction, arrhythmias, heart failure, and other cardiovascular complications (de Boer et al., 2003; Xiao et al., 2023). Xiao et al. (2023) demonstrated that glycine betaine mitigates myocardial hypertrophy in spontaneously hypertensive rats by inhibiting the stromal interaction molecule/ORAI calcium release-activated calcium modulator 1 (STIM1/Orai1) signaling pathway. Elevated STIM1 expression in angiotensin II-induced hypertension is linked to endoplasmic reticulum-induced vascular dysfunction through NADPH oxidase and transforming growth factor-beta (TGF-β) pathways (Kassan et al., 2016; Luo et al., 2024). Treatment with glycine betaine (200 and 400 mg/kg) reduced the systolic and diastolic blood pressure and heart rate. Additionally, betaine decreased the expression of atrial natriuretic peptide and B-myosin heavy-chain in the myocardium (Xiao et al., 2023).

A report from the American Heart Association highlights that nearly all hemodialysis patients experience elevated blood pressure, a condition closely linked to an increased risk of cardiovascular events and mortality (Bansal et al., 2023). A cohort study conducted by Wang et al. in 2018 explored the relationship between serum betaine concentration and hypertension in dialysis patients. The study included 368 patients of varying ages and genders, and found that there is a substantial link between betaine levels and blood pressure, particularly in female subjects (Wang et al., 2018). The results indicated that lower plasma betaine levels were associated with higher blood pressure in the hemodialysis group. These findings indicate that betaine deficiency may play a crucial role in the development of hypertension among dialysis patients.

Building on this research, Huang et al. studied the correlation between serum betaine levels and blood pressure in a larger community-based cohort. Their study revealed that elevated serum betaine levels were connected to lower systolic and diastolic blood pressure, along with reduced pulse pressure (Huang et al., 2023). Furthermore, a nested case–control study was conducted during the China Stroke Primary Prevention Trial (CSPPT). This study included 622 patients with a first stroke. The results revealed a U-shaped association between baseline serum betaine levels and the risk of the first ischemic stroke. Specifically, the risk of the first ischemic stroke decreased with increasing betaine levels up to 77.7 μmol/L. However, further increase of serum betaine (>77.7 μmol/L) increased the risk of ischemic stroke, making a U-shaped association. Interestingly, there was no significant association between serum betaine and the risk of the first hemorrhagic stroke (Xie et al., 2020). Interestingly, the U-shaped relationship observed indicates that the association between betaine and cardiovascular outcomes may not be strictly linear. Moderate increases in circulating betaine were associated with a reduced risk of ischemic stroke, whereas high serum betaine concentrations were associated with an increased risk. The reasons for this observation are unclear, but they may relate to changes in betaine metabolism, renal function, dietary habits, or other confounding cardiometabolic factors, rather than betaine having a direct harmful effect. Nevertheless, these data emphasize the importance of defining an optimal therapeutic range for betaine. Further prospective clinical studies are required before recommending long-term high-dose supplementation.

**FIGURE 4:**
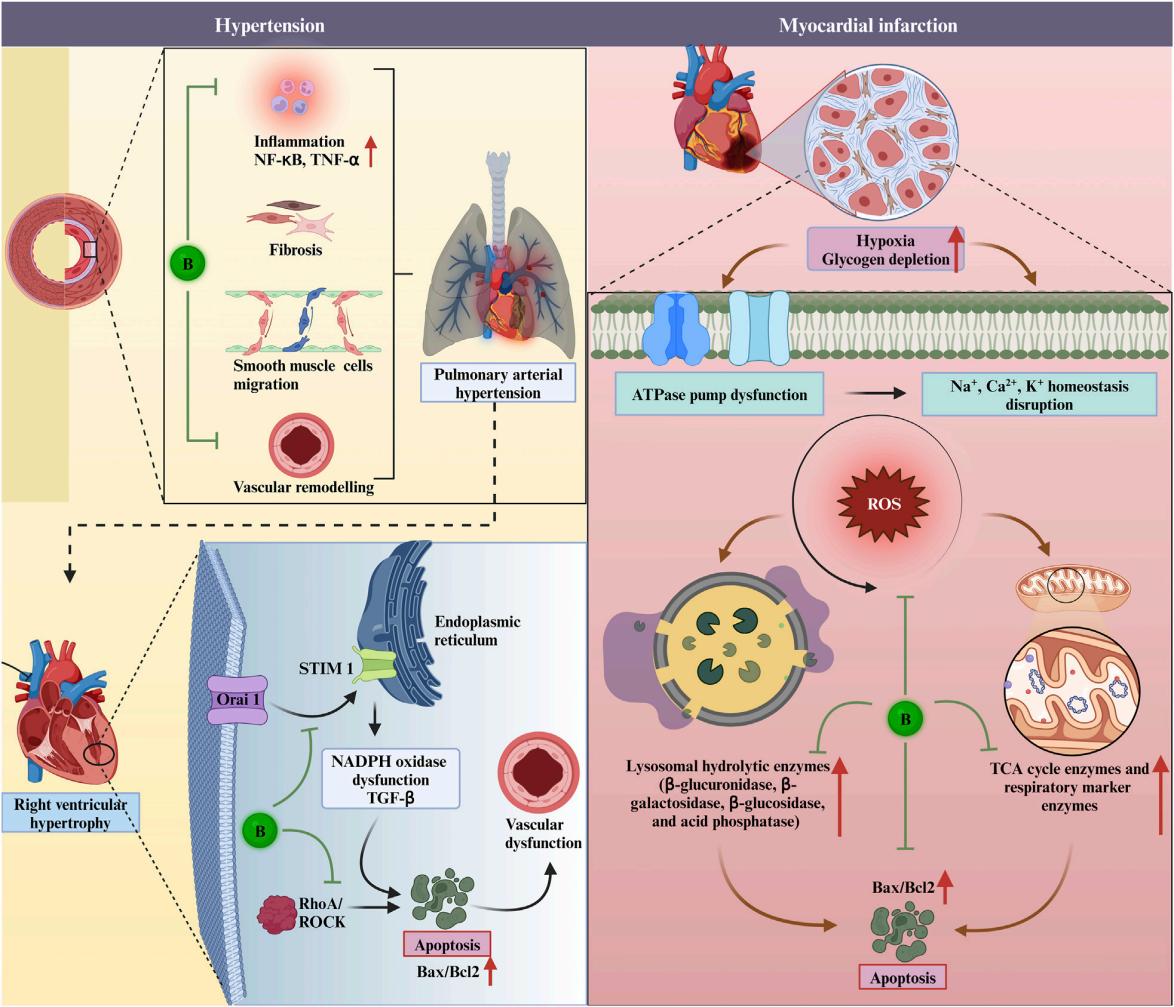
Biological effects of betaine on cardiovascular complications. Betaine (B) reduces pulmonary arterial hypertension by decreasing vascular inflammation, which is achieved through the suppression of pro-inflammatory mediators, including NF-κB and TNF-α. This process also involves the inhibition of smooth muscle-cell migration, fibrosis, and vascular remodeling. These effects are associated with an improvement in pulmonary vascular dysfunction and may slow the progression of right ventricular hypertrophy. Betaine also modulates calcium signaling pathways by decreasing STIM1 and Orai1 activity, which subsequently attenuates endoplasmic reticulum stress, NADPH oxidase-mediated oxidative stress, TGF-β signaling, and ROCK-mediated vascular dysfunction and apoptosis. In the case of myocardial infarction, hypoxia and glycogen depletion due to ischemia cause disruption to ATPase pump activity and intracellular ion homeostasis. Betaine protects Na^+^, Ca^2+^, and K^+^ homeostasis, decreases ROS generation, and attenuates oxidative stress-mediated cellular injury. Furthermore, betaine decreases lysosomal hydrolase activity, modulates the TCA cycle and respiratory enzymes, and reduces apoptosis by regulating Bax/Bcl-2 signaling. This contributes to preserving mitochondrial function and cardiomyocyte integrity. ATPase, adenosine triphosphatase; NADPH, nicotinamide adenine dinucleotide phosphate; NF-κB, nuclear factor kappa B; Orai1, ORAI calcium release-activated calcium modulator 1; RhoA, Ras homolog family member A; ROCK, Rho-associated protein kinase; ROS, reactive oxygen species; STIM1, stromal interaction molecule 1; TCA, tricarboxylic acid; TGF-β, transforming growth factor-β; TNF-α, tumor necrosis factor-α.

Overall, the above studies support betaine’s promising role in managing hypertension and its related complications. Betaine’s ability to lower blood pressure, reduce vascular remodeling, and alleviate myocardial hypertrophy emphasizes its therapeutic value. Nevertheless, the reported U-shaped association between serum betaine levels and ischemic stroke risk indicates that excessively high circulating betaine concentrations may not offer further cardiovascular benefits. The underlying mechanisms are unclear, but these findings emphasize the need to define an optimal therapeutic range and evaluate the long-term safety of betaine supplementation. Further research is essential to fully understand its efficacy and optimize its use, particularly in specialized populations such as dialysis patients. Addressing betaine deficiency and maintaining appropriate levels could be an effective strategy for controlling hypertension and enhancing cardiovascular health (Figure 4).

#### 4.4.4 Betaine’s role in preventing congenital heart disease

Despite advances in the medical-care system, the global burden of congenital heart disease (CHD) remains significantly high. According to the Global Burden of Disease Study, CHD was responsible for approximately 261,247 deaths worldwide in 2017 (van d et al., 2011; Kang et al., 2023). However, a projected study reported that by 2050, there will be approximately 510,000 adult CHD patients in the United States alone (Benziger et al., 2015). Notably, prenatal alcohol exposure is a significant risk factor linked to various CHD subtypes, including endocardial cushion and conotruncal defects (Harvey et al., 2022; Dorsey et al., 2025).

Watanabe et al. (2016) conducted a study using an avian embryo model to evaluate the impact of betaine on congenital anomalies caused by prenatal alcohol exposure. The study showed that supplementing betaine reduced gross structural defects and cardiac conditions such as ventricular septal defects and abnormal atrioventricular valve morphology. Additionally, the diameters of the great vessels and the thickness of the interventricular septum had been restored, indicating that betaine could be an effective preventive measure against alcohol-induced heart defects. Similarly, Karunamuni et al. (2017) found that betaine supplementation improved cardiac development and reduced the incidence of CHD in a prenatal alcohol-exposed rodent model. Their findings indicated that betaine not only increased survival rates but also ameliorated late-stage cardiac defects such as absent vessels and hypertrophic ventricles.

Together, these studies highlight betaine’s protective role in preventing congenital heart defects, especially when there is exposure to harmful prenatal factors such as alcohol. The authors emphasized betaine’s potential as a valuable supplement for reducing the risk of congenital heart anomalies and improving prenatal care strategies. Future studies should explore the mechanistic insights into betaine’s effects on CHD and determine an appropriate betaine supplementation dose.

#### 4.4.5 Betaine’s role in cardiotoxicity

Cardiotoxicity, a severe and often life-threatening side effect, is frequently associated with exposure to certain environmental pollutants and chemotherapeutic agents (Rachma et al., 2024). One of the cardiotoxicants, sodium arsenite (NaAsO_2_), is known to contribute to chronic heart diseases through prolonged exposure to contaminated water. A study by Shariati et al. (2024) explored the protective effects of betaine against NaAsO_2_-induced cardiotoxicity. In their study, mice were exposed to NaAsO_2_ for 8 weeks, leading to significant cardiac damage, along with increased expression of inflammatory markers such as TNF-α and NF-κB. Betaine was administered during the last 2 weeks of the study. The treated mice exhibited reduced oxidative stress and inflammation, leading to a decrease in cardiac injury markers and improved antioxidant enzyme activities (Shariati et al., 2024). In another study, Singh et al. explored the potential of betaine in attenuating DOX-induced cardiotoxicity. In this study, male Sprague-Dawley rats were administered DOX to induce cardiomyopathy, followed by betaine treatment for 28 days. Betaine significantly reduced the level of cardiac injury markers and improved oxidative stress. Histopathological alterations caused by DOX were restored by betaine. On a molecular level, betaine modulated key signaling pathways, including 5′ AMP-activated protein kinase (AMPK), nuclear factor erythroid 2-related factor 2 (Nrf2), and TGF-β, thereby reducing inflammation and fibrosis (Singh et al., 2024). Furthermore, Mohammadpour et al. studied the mechanisms by which betaine alleviates DOX-induced cardiotoxicity, focusing on the NLRP3/SIRT1 pathway. Betaine significantly improved cardiac function by reducing serum levels of cTnI, LDH, and CK-MB. Additionally, it suppressed oxidative stress and inflammatory responses. Importantly, betaine upregulated the expression of SIRT1, a protein crucial in cellular stress responses, and downregulated the NLRP3 inflammasome, thereby reducing inflammation and oxidative stress in cardiac tissue (Mohammadpour et al., 2024).

Overall, these research studies underscore the potential of betaine as a promising cardioprotective agent. Its ability to reduce oxidative stress, inflammation, and fibrosis makes it a valuable therapeutic candidate for preventing and managing cardiotoxicity induced by environmental toxins and chemotherapeutic agents. Future studies should examine the role of betaine in the one-carbon cycle, hyperhomocysteinemia, and cardiotoxicity to better understand its mechanisms beyond its antioxidative, anti-inflammatory, and anti-fibrotic properties.

### 4.5 Betaine prevents the progression and development of diabetes mellitus

Based on recent data from the International Diabetes Federation (IDF), 11.1% or one in nine of the adult population aged between 20 and 79 are living with diabetes. Alarmingly, the data also report that four in 10 people are unaware that they have diabetes. As of 2025, 590 million people suffer from diabetes worldwide. However, projections indicate that by 2050, over 850 million people will have diabetes (IDF, 2025). Insulin resistance and impaired glucose tolerance are the primary characteristics of type 2 diabetes. Prolonged high blood glucose levels can cause severe diabetic complications such as neurological changes, diabetic foot, angiopathies, retinopathies, cardiovascular issues, and renal failure. Therefore, it is crucial to maintain blood glucose levels within the normal range to prevent secondary complications of diabetes (Szkudelska and Szkudelski, 2022).

Circulating betaine levels can be a prognostic predictor of type 2 diabetes mellitus (T2DM), as reduced circulating betaine is linked with an increased risk of developing T2DM (Garcia et al., 2019). Studies in animal models indicate that betaine directly impacts metabolic health. High-fat diets can decrease plasma betaine levels by upregulating genes involved in betaine metabolism, such as BHMT, DMGDH, MAT1A, and phosphatidylethanolamine N-methyltransferase (PEMT). Additionally, betaine has been shown to increase fibroblast growth factor 21 (FGF21) levels and improve metabolic health in mice (Ejaz et al., 2016; Grizales et al., 2018). Moreover, betaine has been found to enhance lipolytic activity in adipose tissue cells, reduce lipid accumulation in skeletal muscle, improve insulin levels, regulate glucose metabolism, and inhibit the release of pro-inflammatory cytokines such as TNF-α, IL-1β, and IL-6 (Islam and Wilson, 2012). Moreover, betaine also regulates the expression of PPARγ coactivator 1α, a critical transcription factor involved in regulating hepatic gluconeogenesis. A study reported that betaine enhances learning and memory abilities in diabetic rats by modulating the PI3K/Akt signaling pathway and reducing p-mTOR expression (Huang et al., 2020). Additionally, Esfahani et al. have demonstrated the efficacy of betaine in ameliorating NaAsO_2_-induced diabetes (10 mg/kg/day for 4 weeks). Co-administration of betaine led to improved glucose intolerance and offered protection against the oxidative and inflammatory liver damage caused by arsenic exposure. Notably, among the tested doses (125 mg/kg, 250 mg/kg, and 500 mg/kg), the 500 mg/kg dosage of betaine displayed the most significant protective effects (Esfahani et al., 2023).

Furthermore, a study showed that betaine could mitigate the adverse effects of hyperglycemia-induced senescence and oxidative damage in ovarian and testicular cells. Betaine-treatment reduced the levels of MDA and methylglyoxal and downregulated aging-related signaling (Mohammadi et al., 2024).

Overall, betaine shows potential as a therapeutic agent for managing type 2 diabetes and its complications due to its roles in metabolism and inflammation, emphasizing its broader implications in both metabolic and reproductive health. Future studies should incorporate betaine as a supplement with anti-diabetic treatment, such as targeting FGF21 and GLP-1, as betaine may play an additive role against T2DM.

### 4.6 Role of betaine in liver diseases

The global burden of chronic liver diseases (CLDs) is increasing day by day. By 2021, the global prevalence was reported to be 1.7 billion, while mortality was reported to be 1.4 million. Furthermore, by 2050, it is projected that the prevalence of CLDs will increase at an average annual rate of 0.20% (Zhao et al., 2025). Devarbhavi et al. recently published findings on the global impact of liver disease, revealing an annual death toll exceeding two million, constituting 4% of global mortality. Notably, one-third of these fatalities occur among females. Liver cancer alone is responsible for 600,000 to 900,000 deaths annually. At present, liver disease ranks as the 11th-leading cause of global mortality, with its true impact often underestimated. Cirrhosis, in particular, stands as the 10th-leading cause of death in Africa, the ninth in Southeast Asia and Europe, and the fifth in the Eastern Mediterranean (Devarbhavi et al., 2023). Betaine has provided evidence of hepatoprotection against various liver diseases (Wang et al., 2021). Apart from direct hepatoprotection, betaine also enhances the diversity of the microbiome and increases the population of bacteria that produce short-chain fatty acids (SCFAs), thereby preventing obesity and glucose intolerance, which are major risk factors of developing hepatic complications (Du et al., 2021).

Interestingly, liver cells highly express Na^+^- and Cl^−^-dependent betaine GABA transporter (BGT-1) that uptake betaine and accumulate it within the hepatocytes (Zhou et al., 2012). Moreover, it has been reported that methyl donor supplementation can alter lipid metabolism (Peng et al., 2023), and lipids are critical players in various CLDs and liver cancer (Paul et al., 2022). Furthermore, supplementation of betaine influences one-carbon metabolism and increases the SAM/SAH ratio and glutathione. Betaine strengthens the tight connection between the epithelial cells of the small intestine, improves intestinal permeability and liver function, and restores histopathological alterations in the liver and intestine by regulating the LPS/TLR4/MyD88 pathway (Chen et al., 2020). In the following subsections, betaine’s role has been described in different liver complications.

#### 4.6.1 Betaine’s role in acute liver failure

Acute liver failure (ALF) is characterized by rapid liver dysfunction in individuals without pre-existing liver disease. A report states that approximately 2.3 billion people consume alcohol, with 40% classified as heavy drinkers. Alarmingly, it has been reported that approximately 90%–95% of heavy drinkers develop ALD (Lee, 2022; Narro et al., 2024).

Khodayar et al. reported that betaine mitigated acetaminophen-induced hepatotoxicity by attenuating oxidative liver injury and modulating Nrf2-related cytoprotective signaling, including HO-1 expression (Khodayar et al., 2020). Furthermore, Heidari et al. evaluated the efficacy of betaine on mitochondrial function and oxidative stress in cases of acute and chronic liver injury induced by bile duct ligation and thioacetamide, respectively. Two doses of betaine (10 mg/kg and 50 mg/kg) were tested in these models. The results indicated that betaine mitigated mitochondrial dysfunction and oxidative stress, thus providing hepatoprotection (Heidari et al., 2018).

Overall, betaine plays critical roles in mitigating liver injury by reducing oxidative stress and enhancing hepatocyte survival. Research highlights the importance of betaine in protecting liver function through mechanisms involving Nrf2, HO-1, and mitochondrial regulation. Thus, betaine presents a promising therapeutic avenue for improving liver health in ALF.

#### 4.6.2 Betaine’s role in metabolic dysfunction-associated steatotic liver disease (MASLD)

**FIGURE 5:**
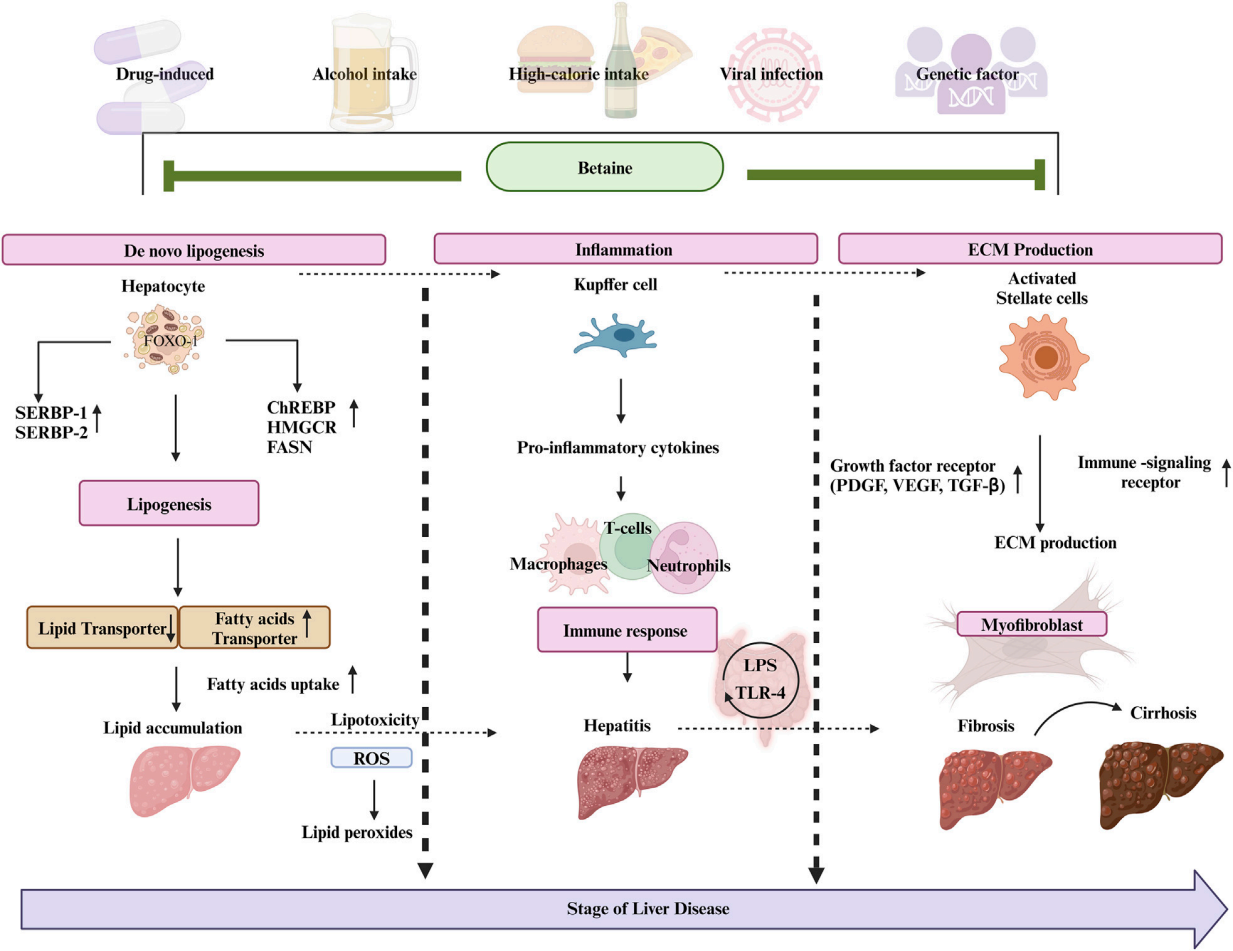
Pathogenesis of liver disease. In hepatocytes, the promotion of *de novo* lipogenesis, lipid accumulation, lipotoxicity, and oxidative stress is driven by the dysregulation of lipogenic transcription factors, such as SREBP-1, SREBP-2, ChREBP, HMGCR, and FASN. These processes stimulate the production of reactive oxygen species (ROS) and lipid peroxidation, which exacerbate hepatic injury. Lipotoxicity and oxidative stress activate Kupffer cells, which secrete more pro-inflammatory cytokines and recruit immune cells, including macrophages, neutrophils, and T-cells. Activation of the LPS/TLR4 pathway exacerbates hepatic inflammation and contributes to the development of hepatitis. Chronic inflammation stimulates hepatic stellate cells (HSCs) and increases the expression of growth factor receptors, such as PDGF, VEGF, and TGF-β, along with the production of ECM and myofibroblast formation. Progressive deposition of ECM leads to fibrosis and cirrhosis. Betaine has hepatoprotective properties, attenuating hepatic lipid accumulation, oxidative stress, inflammatory responses, and fibrogenic signaling. This slows the progression of chronic liver disease. ChREBP, carbohydrate-responsive element-binding protein; ECM, extracellular matrix; FASN, fatty acid synthase; FOXO1, forkhead box protein O1; HMGCR, 3-hydroxy-3-methylglutaryl-CoA reductase; LPS, lipopolysaccharides; PDGF, platelet-derived growth factor; ROS, reactive oxygen species; SREBP, sterol regulatory element-binding protein; TGF-β, transforming growth factor beta; TLR-4, toll-like receptor 4; VEGF, vascular endothelial growth factor.

Steatotic liver disease is an umbrella condition that encompasses a range of conditions associated with fatty liver. Among all liver conditions, MASLD accounts for more than 32% of patients (Teng et al., 2023). MASH, a progressive form of MASLD, is linked with cardiometabolic risk factors, including T2DM, obesity, and hypertension, among others (Dufour et al., 2021). Fat accumulation in the liver leads to lipotoxicity, leading to hepatocellular inflammation, fibrosis, and cellular injury. If MASH is left untreated, it might further progress to cirrhosis, where extensive scarring disrupts the liver’s normal structure and function, potentially leading to liver failure and hepatocellular carcinoma (Figure 5) (Wei et al., 2024; Parola and Pinzani, 2024; Yadav et al., 2022). A study by Seo et al. explored the effects of betaine on diet-induced MASLD and its interplay with apoptosis, lipid metabolism, and autophagy. The mice were fed a research diet for 7 days, followed by supplementation with 0.2%, 0.5% and 1% betaine in their drinking water for 1 week. The results indicated that betaine caused substantial changes in steatosis, liver damage, oxidative stress, and the AMPK pathway (Seo et al., 2024). Furthermore, studies have identified the association between gut microbiota and the progression of MASLD (Aron-Wisnewsky et al., 2020; Jayachandran and Qu, 2023). Interestingly, Sun et al. fed a high-fat diet (HFD) to female mice for 4 weeks, and then the mice were mated. Betaine at 1% was supplemented during pregnancy and lactation, while HFD was continued until their offspring were 3 weeks old. The offspring were fed a standard diet until 10 weeks of age. The study found that maternal overweight and obesity affect the gut microbiome of their offspring at birth. However, betaine supplementation restored hepatic lipid accumulation, elevated triglycerides, increased total cholesterol, and dysregulated mRNA expression of genes associated with lipid oxidation (PPARα and CPT1α) and transport protein (FATP2). Betaine supplementation also improved the gut microbiota and short-chain fatty acids (SCFAs) in the offspring mice (Sun et al., 2023).

Chen et al. evaluated the role of betaine in both *in vitro* and *in vivo* models. In an *in vitro* study, HepG cells were treated with oleic acid (0.2 mM) and betaine (20 mM–160 mM). The findings indicated that betaine elevated the expression of FGF10 and AMPK, which play a role in restoring fatty acid accumulation. In the *in vivo* study, apolipoprotein E-deficient (*Apoe* ^−/−^) mice were fed a HFD and supplemented with or without 2% betaine in their drinking water. The findings demonstrated betaine’s protective effect at the biochemical, histopathological, and molecular levels. Overall, the study indicated that betaine may mitigate MASLD by targeting *de novo* lipogenesis, fatty acid oxidation, and hepatic lipid accumulation (Chen et al., 2021). Furthermore, Huang et al. used transcriptomic and translational analyses to determine betaine’s effectiveness against MASLD. They reported that betaine reduced the expression of IDI1, CYP51A1, TM7SF2, and APOA4, which are involved in lipid biosynthesis (Huang et al., 2021). These studies highlight the potential of betaine in regulating dyslipidemia in MASLD.

A case–control study conducted by Sookoian et al. included biopsy-diagnosed MASLD and MASH patients. The study found that lower betaine levels are associated with increased MASLD severity. Moreover, this study reported that betaine levels were lower in MASH patients than in MASLD patients, indicating a strong association between betaine and the progression of MASLD to MASH. Additionally, individuals with more advanced MASLD/MASH have mutations in dimethylglycine dehydrogenase, a mitochondrial enzyme that plays a crucial role in choline metabolism. This mutation leads to an alteration in choline metabolism, ultimately decreasing betaine production (Sookoian et al., 2017). FoxO6 and PPARγ are the key factors that play an important role in lipid metabolism. In 2024, Kim et al. explored the effect of betaine on HepG2 cells and liver tissues from *db/db* mice treated with betaine. The study found that betaine decreased lipid accumulation and lipogenesis-related gene expression induced by FoxO6. Moreover, betaine also improved hepatic insulin signaling and inhibited the FoxO6–PPARγ interaction, leading to lowering of triglyceride levels in hepatic cells. In the *db/db* mice, oral administration of betaine significantly ameliorated hepatic steatosis by decreasing PPARγ levels. Thus, this study confirmed that betaine inhibits lipogenic gene transcription through the suppression of FoxO6 and PPARγ interaction (Kim et al., 2022). A recent study by Xie et al. used oleic acid and a HFD to induce steatotic models in HepG2 cells and C57BL/6 mice, respectively. Oleic acid and HFD suppress the expression of miR-96-5p, thus leading to increased lipogenic gene expression and lipid droplet accumulation. Betaine treatment at a dose of 120 mg/kg/day through oral gavage substantially elevated the expression of miR-96-5p and lowered the triglyceride level. In hepatocytes, insulin-like growth factor-1 receptor (IGF1R) augments lipogenesis through the SREBP-1c pathway. Interestingly, Xie et al. (2025) reported that elevated expression of miR-96-5p directly targeted IGF1R and decreased its expression, ultimately decreasing lipogenesis.

Overall, these studies demonstrate the promising therapeutic potential of betaine in fatty liver disease. Nevertheless, its efficacy in advanced MASH, characterized by severe hepatocellular ballooning and progressive fibrosis, remains insufficiently explored. Notably, emerging evidence suggests a potential interaction between betaine and incretin-related pathways. Future studies should investigate whether betaine modulates GLP-1 secretion, GLP-1 receptor (GLP-1R) expression, or downstream GLP-1R signaling, as these mechanisms may contribute to its protective effects in advanced MASH.

#### 4.6.3 Betaine’s role in alcoholic liver disease

Alcohol is the most commonly abused substance worldwide, leading to alcoholic liver disease. Chemokines, ROS, and other pro-inflammatory cytokines are byproducts of alcohol metabolism that are toxic to tissues and organs (Dukić et al., 2023). Acetaldehyde, a reactive and toxic compound, is produced during alcohol metabolism in hepatocytes and is broken down to acetate by the action of acetaldehyde dehydrogenase (Lieber, 1988). Cytochrome P4502E1 is also involved in alcohol metabolism, which enhances the generation of ROS and inflammation. The microsomal ethanol-oxidizing system (MEOS), discovered by C. S. Lieber and L. M. DeCarli in the mid-20th century, accelerates alcohol metabolism, leading to faster damage to tissues and organs (Gouillon et al., 2000; Ye et al., 2012). Hepatocytes, Kupffer cells, monocytes, neutrophils, T-lymphocytes, and hepatic stellate cells also play a role during ALD pathogenesis by producing inflammatory cytokines and chemokines. Alcohol consumption also causes gut dysbiosis and disrupts the gut–liver axis, leading to further liver damage (Xu et al., 2014; Marin et al., 2017). Alcohol consumption reduces endogenous hepatic betaine and SAM levels, increases SAH and plasma homocysteine levels, and inhibits methionine synthase activity, leading to a decreased SAM:SAH ratio (Purohit et al., 2007; Kempson et al., 2013). Betaine has a protective effect in alcohol-induced liver disease by boosting SAM and glutathione synthesis, decreasing homocysteine and SAH levels, and improving lipid export from the liver (Purohit et al., 2007). Additionally, betaine, by restoring the SAM levels, mitigates endoplasmic reticulum stress and reduces the accumulation of damaged proteins, steatosis, and apoptosis in liver cells (Thomes et al., 2015).

It is reported that betaine can reduce accumulation of hepatic triglyceride by acting as a methyl group donor, which is crucial for the methionine cycle in the liver. This lipotropic characteristic helps restore methionine homeostasis and repair the alcohol-induced reduction in methylation potential. Consequently, betaine prevents hepatic lipid accumulation and improves liver health (Day and Kempson, 2016). Chronic alcohol consumption leads to hypomethylation and increases lipolysis in adipose tissue. Betaine has shown hepatoprotective effects in ALD animal models by enhancing adipose tissue function and methylation status, which in turn reduces liver damage (Osna et al., 2017).

Furthermore, in 2007, Zhenyuan et al. demonstrated that prolonged alcohol exposure leads to an abnormal accumulation of homocysteine in adipose tissue, leading to decreased adiponectin levels. Their study revealed that betaine supplementation significantly reduced homocysteine levels, thereby restoring adiponectin levels and mitigating the adverse effects of alcohol on liver function (Zhenyuan et al., 2008). In a study conducted by Yang et al., the effects of betaine on alcohol-induced hepatic lipid metabolic disorders in rats were analyzed. The study revealed that betaine supplementation prevented the increase in hepatic triglycerides, free fatty acids, and cholesterol levels induced by alcohol consumption. Furthermore, betaine downregulated the expression of lipogenic genes such as *DGAT1*, *DGAT2*, *SREBP-1c*, *HMG-CoA*, and *FAS*, while upregulating *PGC-1α*, a crucial regulator of lipid metabolism. These findings indicate that betaine helps maintain lipid homeostasis in the liver, thereby protecting against alcohol-induced hepatic steatosis (Yang et al., 2017). Overall, betaine emerges as a promising therapeutic agent in the management of ALD, offering protection through various mechanisms. Betaine modulates methylation processes, improves lipid metabolism, and reduces oxidative stress. Therefore, betaine is a crucial compound in the fight against ALD. By addressing the metabolic disruptions caused by chronic alcohol consumption, betaine not only prevents the progression of liver damage but also promotes overall liver health.

### 4.7 Role of betaine in kidney diseases

Chronic kidney disease (CKD) is characterized by a progressive decline in kidney function over time due to conditions such as diabetes and hypertension. In contrast, acute kidney injury (AKI) is a sudden and severe decrease in kidney function, often triggered by reduced blood flow to the kidneys, infections, or nephrotoxicity (Yang et al., 2011). Patients with CKD, specifically end-stage renal disease (ESRD), often experience various issues with amino acid metabolism. One such issue is an elevated level of homocysteine, a sulfur-containing amino acid in the plasma, leading to a gradual decline in renal function (Mallamaci et al., 2002).

Numerous studies have demonstrated betaine’s antioxidant properties in kidney disease. Ozturk et al. studied betaine’s potential protective effects against kidney damage induced by carbon tetrachloride (CCl_4_). In rats treated with CCl_4_, betaine reduced oxidative stress by restoring activities of SOD, catalase, and glutathione peroxidase (GPx) levels. Betaine pre-treatment at a dose of 830 mg/kg for 11 days prevented the glomerular and tubular changes in the renal cortex caused by CCl_4_ exposure, indicating that betaine alleviates the nephrotoxic effects of CCl_4_ (Ozturk et al., 2003). Betaine serves as a crucial osmoprotectant in the kidneys, as it is transported by BGT1, which is abundant in the renal medulla and regulated by osmotic stress. In patients with chronic kidney disease, betaine levels decrease as kidney failure progresses, with lower levels in severe stages (Guo et al., 2021). Betaine also exerts a protective effect against cadmium-induced kidney damage, as reflected by reduced serum creatinine and blood urea nitrogen levels. Moreover, betaine protected the kidneys by inhibiting inflammatory cytokine production and decreasing lipid peroxidation by elevating antioxidant enzyme levels (Hagar and Al Malki, 2014).

In 2019, Ghartavol et al. analyzed the effect of betaine on renal function in isoproterenol-induced myocardial infarction. Betaine at different doses (50, 150, and 250 mg/kg) was administered for 60 days. After the injection of isoproterenol, the rats were examined for the expression of inducible nitric oxide synthase, TNF-α, serum creatinine levels, and histopathological analysis. The study found that betaine showed a protective effect by decreasing the expression of nitric oxide synthase and TNF-α (Ghartavol et al., 2019).

**FIGURE 6:**
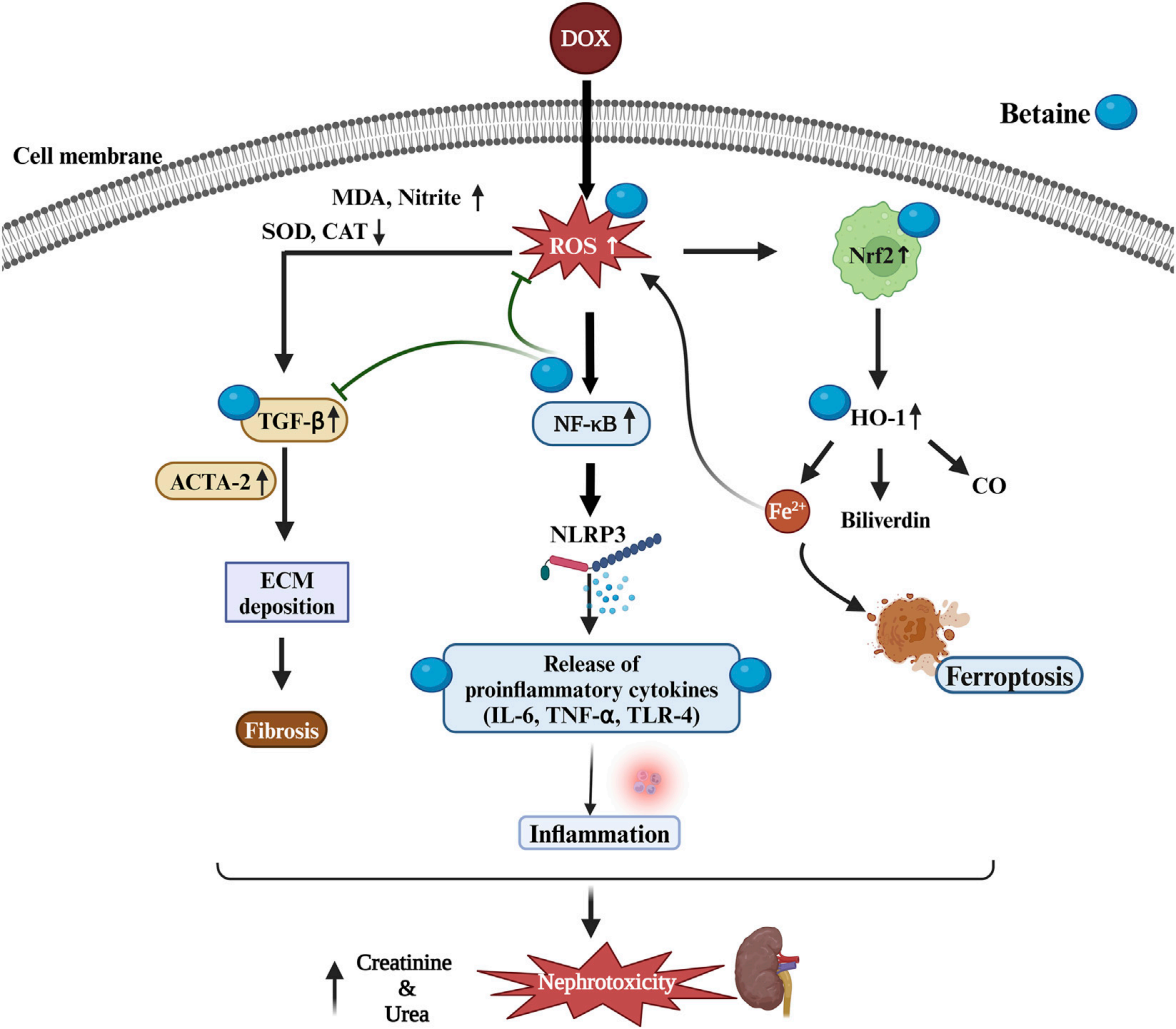
Protective role of betaine against doxorubicin (DOX)-induced nephrotoxicity. DOX administration promotes the excessive generation of ROS, leading to oxidative stress, inflammation, fibrosis, and renal dysfunction. Increased ROS production is associated with elevated malondialdehyde (MDA) and nitrite levels, accompanied by decrease in the activity of endogenous antioxidant enzymes such as superoxide dismutase (SOD) and catalase (CAT). Oxidative stress activates the NF-κB signaling pathway and the NLRP3 inflammasome, leading to increased production of pro-inflammatory mediators, including IL-6 and TNF-α, and associated inflammatory responses. ROS-mediated injury also stimulates the expression of TGF-β and alpha-smooth muscle actin (ACTA-2), promoting ECM deposition and renal fibrosis. Furthermore, oxidative stress influences Nrf2/HO-1 signaling, which regulates cellular antioxidant defense mechanisms. Betaine mitigates the effects of oxidative stress by reducing ROS generation, suppressing NF-κB/NLRP3-mediated inflammation, decreasing fibrogenic signaling, and modulating Nrf2/HO-1 activity. This protects against DOX-induced nephrotoxicity and improves renal function. ACTA-2, alpha-smooth muscle actin; CAT, catalase; CO, carbon monoxide; DOX, doxorubicin; ECM, extracellular matrix; Fe^2+^, ferrous iron; HO-1, heme oxygenase-1; IL-6, interleukin-6; MDA, malondialdehyde; NF-κB, nuclear factor-kappa B; NLRP3, NOD-like receptor protein 3; Nrf2, nuclear factor erythroid 2–related factor 2; ROS, reactive oxygen species; SOD, superoxide dismutase; TGF-β, transforming growth factor-beta; TLR-4, toll-like receptor 4; TNF-α, tumor necrosis factor-α.

Furthermore, al-Hafyan et al. analyzed the beneficial effects of betaine on arsenic-induced hepatic and renal injuries. Betaine was administered as 2% of the diet. The study revealed a significant reduction in endogenous antioxidant levels along with an increase in MDA level in the arsenite-administered rats. Interestingly, betaine treatment significantly restored the antioxidant–oxidant balance in both tissues. The study indicates that betaine could be a potential candidate for mitigating arsenic-induced toxicity (al-Hafyan et al., 2023). Similarly, Sharma et al. (2022) and Norouzzadeh et al. (2024) independently reported that betaine treatment prevented arsenic-induced alterations. Betaine significantly improved renal architecture and mitigated the reduction in renal endothelial nitric oxide synthase expression, thus attenuating arsenite-induced nephrotoxicity (Sharma et al., 2022; Norouzzadeh et al., 2024). Moreover, Farid Abdelrazek et al. analyzed the preventive effect of glycine betaine on lead-induced renal and hepatic damage in male albino rats. Lead administration caused a significant decline in renal function, including a 17.4% and 23.7% increase in creatinine and urea levels, along with increased liver function parameters. Treatment of glycine betaine in lead-intoxicated rats caused a substantial decline in lipid peroxidation, ALT, AST, creatinine, and urea levels, along with an increase in anti-oxidant enzymes (Abdelrazek et al., 2022). Recently, Patel et al. reported the nephroprotective potential of betaine in doxorubicin-induced nephrotoxicity (Figure 6). Betaine decreased the creatinine and urea levels, along with the restoration of histopathological alterations. Mechanistically, betaine upregulated the Nrf2/HO-1 gene expression and prevented inflammation and fibrosis by reducing the NLRP3, TLR-4, TNF-α, IL-6, TGF-β, and α-smooth muscle actin expression in doxorubicin-treated rats (Patel et al., 2023).

Moreover, Jorgačević et al. (2022) examined betaine’s role in modulating macrophage migration inhibitory factor (MIF)-mediated oxidative stress, inflammation, and fibrogenesis in thioacetamide (TAA)-induced kidney toxicity using wild-type and MIF knockout C57BL/6 mice. The results showed that after 8 weeks, *mif* ^−/−^ mice had decreased oxidative stress markers (MDA, IL-6, TNF-α, TGF-β1, and PDGF-BB) and increased antioxidant activities compared to that in TAA (200 mg/kg)-administered mice. Betaine (2% w/v dissolved in drinking water) treatment also reduced oxidative stress and cytokine levels, indicating that it enhances the kidney’s antioxidative capacity and reduces inflammation and fibrogenesis (Jorgačević et al., 2022). Furthermore, Hagar et al. analyzed betaine’s protective effects against cisplatin-induced nephrotoxicity. Prophylactic betaine supplementation (250 mg/kg/day) for 21 days before cisplatin administration improved the kidney function, restored antioxidant levels, and reduced inflammatory and apoptotic factors TNF-α and NF-κB and caspase-3 (Hagar et al., 2015).

Liu et al. demonstrated the role of betaine in regulating serum uric-acid levels and renal function in potassium oxonate-induced hyperuricemic mice. Betaine restored the levels of urate transporters (URAT1, GLUT9, OAT1, and ABCG2) in the kidney and enhanced urate excretion. Furthermore, betaine decreased beta-2-microglobulin in urine and upregulated the levels of organic cation/carnitine transporters (OCT1, OCTN1, and OCTN2) in the kidney (Liu et al., 2014).

Another study reported the protective effect of betaine (1% in diet) on ovalbumin (OVA)-induced asthma-associated impaired kidney function in mice. The results show that betaine improves airway inflammation and increases the antioxidant levels in kidney tissue (Pourmehdi et al., 2020). Furthermore, Salahi et al. analyzed betaine’s (1.5% w/w in diet) protective effect on liver and renal function in an animal model of STZ-induced diabetes in pregnant rats. Betaine treatment restored HbA1c, liver function, kidney biomarkers, and histopathological alterations in both tissues in diabetic rats (Salahi et al., 2020). Fan et al. reported betaine’s nephroprotective effect against high-fructose-induced kidney dysfunction, including inflammation, insulin resistance, and dyslipidemia. Moreover, betaine protected against kidney dysfunction and tubular injury by restoring glucose transporter 9, inflammatory cytokine expression, and reducing lipid accumulation through the PPARα pathway (Fan et al., 2014).

Hussein et al. induced nephrotoxicity through cisplatin administration, leading to severe renal damage, increased levels of creatinine and urea, and upregulated expression of miRNA 34a, phospho-p53, caspase 3, cytochrome c, NF-κB, and IL-1β. However, co-administration of betaine significantly reduced these markers and improved renal-tissue integrity. The study concludes that betaine mitigates cisplatin-induced nephrotoxicity by modulating miRNA 34a, apoptosis, and inflammation (Hussein and Al-Dalain, 2021).

**FIGURE 7:**
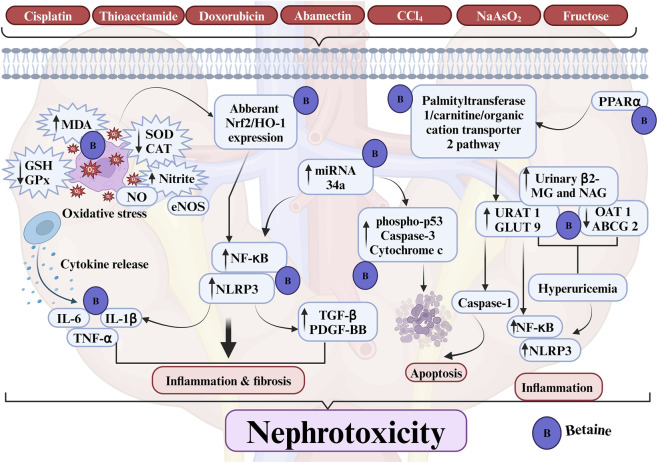
Protective role of betaine against drug/toxin/chemical-induced nephrotoxicity. Various nephrotoxic agents, including cisplatin, thioacetamide, doxorubicin, abamectin, carbon tetrachloride (CCl_4_), sodium arsenite, and fructose, induce renal injury via oxidative stress, inflammation, fibrosis, apoptosis, and disturbances in uric acid metabolism. Nephrotoxicity is characterized by increased lipid peroxidation and elevated levels of nitrite and nitric oxide, along with the depletion of endogenous antioxidants such as GSH, GPx, SOD, and CAT. These alterations contribute to oxidative stress and the dysregulation of the Nrf2/HO-1 pathway. Oxidative stress also activates NF-κB signaling and the NLRP3 inflammasome, leading to the production of pro-inflammatory cytokines such as IL-1β, IL-6, and TNF-α. Persistent inflammation promotes fibrogenic signaling through TGF-β and PDGF, leading to renal inflammation and fibrosis. Additionally, nephrotoxic insults can induce apoptosis via the activation of the phospho-p53, caspase-3, and cytochrome c pathways by microRNA-34a. Alterations in urate transporters, including URAT1, GLUT9, OAT1, and ABCG2, contribute to hyperuricemia and subsequent renal inflammation. Betaine exerts renoprotective effects by attenuating oxidative stress, restoring antioxidant defense systems, suppressing NF-κB/NLRP3-mediated inflammatory responses, reducing apoptosis and fibrogenic signaling, regulating metabolic pathways associated with peroxisome proliferator-activated receptor alpha (PPARα), and modulating renal urate transporters. This protects against nephrotoxicity. ABCG2, ATP-binding cassette subfamily G member 2; ACTA-2, alpha-smooth muscle actin; CAT, catalase; CO, carbon monoxide; DOX, doxorubicin; ECM, extracellular matrix; eNOS, endothelial nitric oxide synthase; Fe^2+^, ferrous ion; GLUT9, glucose transporter 9; HO-1, heme oxygenase 1; IL-6, interleukin 6; NF-κB, nuclear factor kappa-light-chain-enhancer of activated B cells; NLRP3, NOD-like receptor pyrin domain-containing 3; Nrf2, nuclear factor erythroid 2-related factor 2; OAT1, organic anion transporter 1; PDGF-BB, platelet-derived growth factor-BB; PPAPα, peroxisome proliferator-activated receptor alpha; ROS, reactive oxygen species; SOD, superoxide dismutase; TGF-β, transforming growth factor beta; TLR-4, toll-like receptor 4; TNF-α, tumor necrosis factor-alpha; URAT1, urate transporter 1.

One study compared the protective effects of betaine and melatonin, administered alone or in combination, on cisplatin-induced nephrotoxicity in mice. Daily administration of betaine (200 mg/kg) or melatonin (10 mg/kg) significantly mitigated cisplatin-induced alterations. Interestingly, the combination of these two drugs provided far greater protection than when administered alone (Al Za’abi et al., 2021). In summary, these studies provide strong evidence of the nephroprotective effect of betaine against drug/toxins and metabolic-induced renal damage by modulating oxidative stress, inflammation, and fibrosis (Figure 7).

### 4.8 Betaine as an epigenetic regulator

**FIGURE 8:**
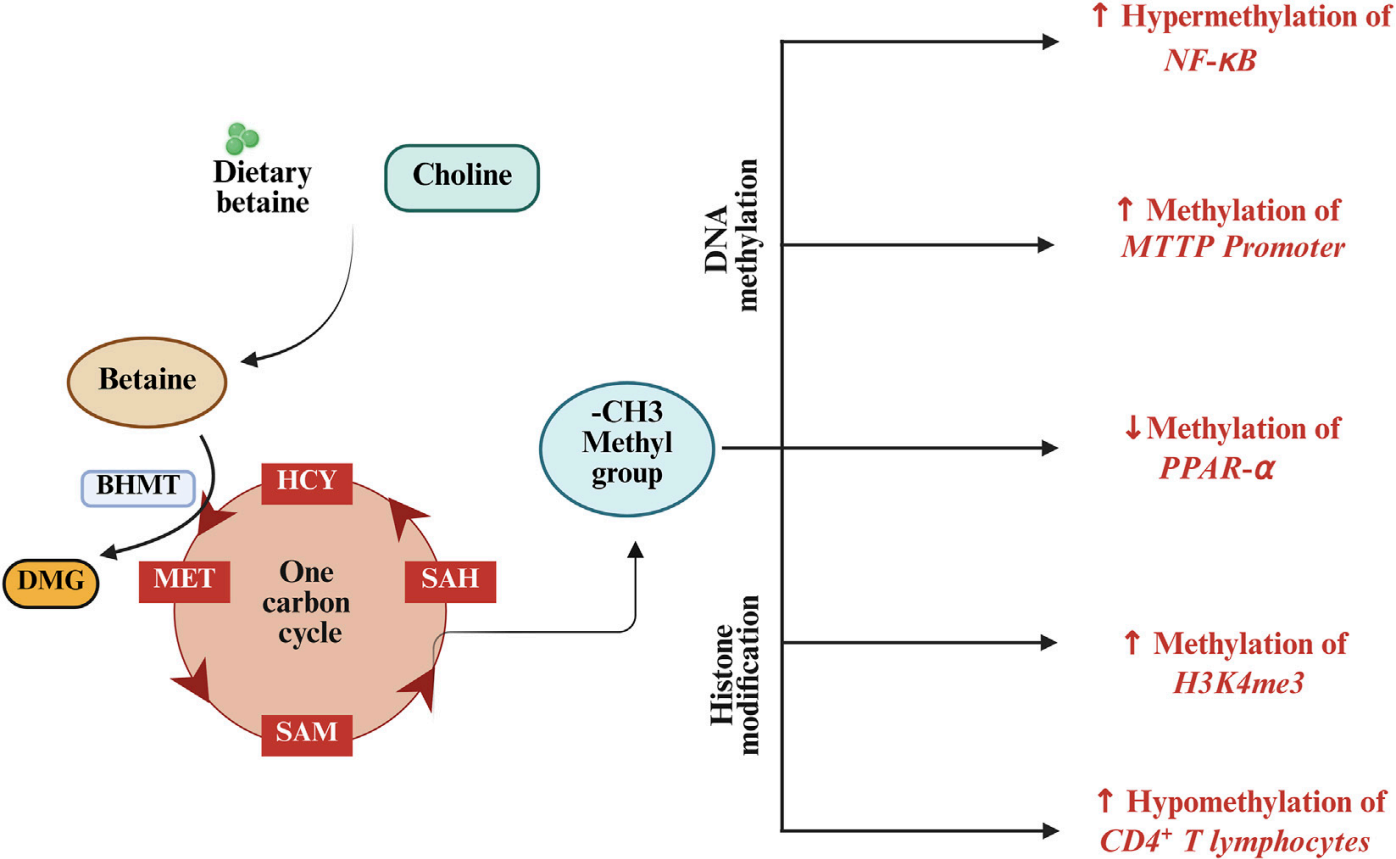
Biochemical effect of betaine through SAM and one-carbon cycle. Dietary betaine and choline are important for one-carbon metabolism. In this process, betaine acts as a methyl donor, facilitating the BHMT-mediated remethylation of homocysteine to methionine and producing DMG as a by-product. Methionine then enters the methionine cycle, where it is converted to SAM (S-adenosylmethionine), the universal methyl donor. After a series of methyl-group transfer reactions, SAM is converted to SAH, which is then regenerated into homocysteine, thus completing the cycle. The methyl groups generated through this pathway participate in epigenetic regulation by modulating DNA methylation and histone modifications. Betaine influences the methylation status of genes involved in inflammation, lipid metabolism, and immune regulation through these mechanisms, including the hypermethylation of NF-κB, increased methylation of the MTTP promoter, decreased methylation of PPAR-α, increased H3K4me3, and altered DNA methylation patterns in CD4^+^ T-lymphocytes. BHMT, betaine-homocysteine methyltransferase; CD4^+^, cluster of differentiation 4 positive; DMG, dimethylglycine; H3K4me3, histone H3 lysine 4 trimethylation; HCY, homocysteine; MET, methionine; MTTP, microsomal triglyceride transfer protein; NF-κB, nuclear factor kappa B; PPAR-α, peroxisome proliferator-activated receptor alpha; SAH, S-adenosylhomocysteine; SAM, S-adenosylmethionine.

Epigenetic processes include DNA methylation, post-translational modifications of histones, and regulation of non-coding RNAs, such as microRNAs. Among these, histone (lysine) methylation and DNA methylation at cytosine–guanine (CpG) sites are well-studied epigenetic processes that control gene expression. These epigenetic pathways are involved in many diseases, including cancer, diabetes, obesity, MASH, and other inflammatory conditions (Zhang, 2018). Betaine is one of the major methyl donors in the one-carbon cycle (Figure 8). Betaine primarily exerts epigenetic effects through its central role in one-carbon metabolism. Through BHMT-mediated remethylation, betaine contributes to the synthesis of SAM. SAM is then converted into SAH, which acts as a potent inhibitor of methyltransferases. Decreased SAM/SAH ratios promote global hypomethylation and aberrant gene expression. However, betaine can restore the methylation potential, which may normalize DNA and histone methylation patterns (Jaiswal et al., 2023). DNA methylation is dynamically regulated by DNA methyltransferases (DNMTs) and demethylating enzymes, including the ten-eleven translocation (TET) family of dioxygenases. This process utilizes the methyl groups provided by the donors such as betaine. DNA methylation is a well-known epigenetic modification that influences gene expression, impacting various biological processes (Li and Zhang, 2014; Williams and Schalinske, 2007). By modulating DNA and histone methylation, betaine can affect the transcription of genes involved in inflammation, lipid metabolism, oxidative stress, and fibrosis. Consequently, betaine-mediated epigenetic regulation may impact key signaling pathways, including NF-κB, the NLRP3 inflammasome, lipid transport, and fatty acid oxidation. Furthermore, the body produces betaine from choline in addition to dietary intake to maintain this methylation balance and epigenetic modifications, if needed (Ashtary-Lark et al., 2022).

Betaine has been shown to increase the expression of transcription factors required for oligodendrocyte development, such as SRY-Box Transcription Factor 10 (SOX10) and NK2 homeobox 2 (NKX-2.2), under oxidative stress. Oligodendrocyte development involves changes in DNA and histone methylation, indicating a role of betaine in the regulation of these genes (Fujita et al., 2016; Sternbach et al., 2021). Another study shows that betaine preserves neuronal mitochondria in the cuprizone mouse model of multiple sclerosis via epigenetic regulation, maintaining the methylation potential (SAM/SAH ratio) under oxidative stress. This process also leads to an increase in methylation levels of H3K4me3, improved neuronal respiration, and prevents axonal damage (Singhal et al., 2020). Additionally, betaine has been found to hypermethylate the NF-κB promoter, which inhibits the release of nitric oxide (NO) from microglia in the brain following LPS administration (Amiraslani et al., 2012).

Wang et al. demonstrated that betaine ameliorated HFD-induced MASLD by increasing genomic methylation and decreasing methylation of the microsomal triglyceride transfer protein (MTTP) promoter. The study reported that HFD leads to loss of choline and SAM, ultimately disabling the methylation processes. In addition, it was found that HFD increases the methylation level of the MTTP gene promoter and DNMT-1 expression (Wang et al., 2014). Betaine decreases DNA methylation of the MTTP promoter. This leads to enhanced expression of MTTP mRNA and protein, which further strengthens the role of MTTP in VLDL assembly and, finally, improves hepatic steatosis through the transport of triglycerides as VLDL-TG (Wang et al., 2014). By reducing the DNA methylation of the PPARα promoter and upregulating its gene expression, betaine supplementation may reduce hepatic triglyceride buildup and oxidative stress (Wang L. et al., 2013). Additionally, betaine lowers oxidative stress by lowering TNF-α and IL-6, reestablishing the equilibrium between oxidants and antioxidants, and modifying the expression of genes linked to apoptosis. Moreover, in methionine–choline deficiency-induced fatty liver disease, betaine promotes cytoprotective Akt/mTOR signaling and modifies the autophagy process. (Veskovic et al., 2019).

It has also been reported that betaine intake during pregnancy was inversely associated with methylation of the IGF2 gene’s differentially methylated regions. On the other hand, maternal choline intake was significantly linked to methylation of the CRH promoter in cord blood. Given that the IGF pathway plays a role in promoting fetal growth, these connections may explain the lower birth weight and cortisol levels in newborns whose mothers consumed higher levels of these nutrients. The methylation status of IGF-2 is crucial for placental and fetal growth since it is an imprinted gene (St-Pierre et al., 2012). Global and localized hypomethylation in CD4^+^ T-cells has been linked to systemic lupus erythematosus (SLE), while hypomethylation in B-cells is linked to the onset of SLE (Renaudineau and Youinou, 2011; Pesqueda-Cendejas et al., 2023). In individuals with SLE, betaine reduces inflammation by modulating the NF-κB signaling pathway and inhibiting the NLRP3 inflammasome (Pesqueda-Cendejas et al., 2023). Furthermore, research has shown that individuals with Crohn’s disease and ulcerative colitis exhibit DNA hypomethylation in various cell types, including immune cells and intestinal epithelial cells. This hypomethylation may contribute to the progression of the diseases, leading to genomic instability and altered gene expression. Betaine, as a methyl donor, can counteract this hypomethylation in these disease conditions (Marangoni et al., 2023).

In a rat model, the administration of betaine through diet has been shown to mitigate neointima formation and vascular stenosis following arterial intimal injury by downregulating S100A9, Serpinh1, and ILF3, while upregulating Hspg2, Fmod, Fbln-5, and LOX proteins through methylation (Jiang et al., 2024). Dietary betaine (0.1% in the basal diet) prevents corticosterone-induced hepatic cholesterol accumulation by modulating DNA methylation, specifically increasing methylation at the *HMGCR* gene and decreasing it at the *CYP7A1* gene (Wu et al., 2024). Oligoasthenozoospermia is the most common semen abnormality in male infertility. In a rat model of oligoasthenozoospermia induced by *Tripterygium wilfordii* glycosides (TWPs), betaine administered at doses of 200, 400, and 800 mg/kg significantly restored testicular damage by significantly increasing the methylation level of spermatogenesis-related genes (Lin et al., 2024). Additionally, administering betaine (2.5% w/v) via drinking water for 14 days inhibited the NLRP3/caspase-1/GSDMD signaling pathway through methylation, leading to the formation of unstable mRNAs. This inhibition further suppressed microglial pyroptosis, ultimately alleviating homocysteine-induced cognitive impairment (Yang et al., 2024).

Betaine has shown promising results when used as a methyl donor and an epigenetic modulator in various disease models. However, the quality of the evidence varies across studies. While some studies have provided direct evidence of changes in DNA or histone methylation following betaine administration, in most cases, the observed epigenetic changes are associative and do not establish a direct causal link between methylation modifications and disease improvement. Furthermore, the specific genomic regions and molecular targets affected by betaine remain unclear in several diseases. Future studies involving genome-wide methylation profiling and mechanistic validation are needed to identify the direct epigenetic targets of betaine and elucidate the extent to which its therapeutic effects are mediated through epigenetic regulation.

## 5 Betaine intervention in clinical studies

We found 61 clinical trial reports on ClinicalTrials.gov. After examining each clinical study report, we discovered that 33 trials involving betaine had been completed, as shown in Supplementary Table S1. Additionally, there are ongoing clinical trials utilizing various forms of betaine (11), while some trials have been withdrawn or have an unclear status (9), as shown in Supplementary Tables S2 and S3, respectively. Betaine is commonly used as a dietary supplement and for digestive aids such as betaine hydrochloride. Research has shown that the highest dose of betaine (4,500 mg) is the most effective in re-acidifying gastric pH to baseline levels in the presence of food (Surofchy et al., 2019). Furthermore, studies have demonstrated that betaine can impact the structure of intestinal villi in the duodenum, jejunum, and ileum. Excessive salt stress can decrease gut villi heights, while betaine administration has been shown to increase villus heights (Guilliams and Drake, 2020). Additionally, higher concentrations of betaine in plasma are associated with lower homocysteine levels in individuals with CVD during fasting. Supplementing with betaine at a dose of 6 g/day for 6–12 weeks has been shown to decrease fasting total homocysteine concentration in healthy volunteers with normal levels by up to 20% compared to that with a placebo after methionine loading by 20%–40% (Olthof and Verhoef, 2005). For individuals with inherited deficiencies in the enzymes responsible for homocysteine metabolism, such as reduced cystathionine beta synthase activity, betaine is considered a safe and effective treatment to lower homocysteine levels (Olthof and Verhoef, 2005). Additionally, research indicates that dietary choline levels may influence plasma homocysteine concentrations (Setoue et al., 2008). A study by Setoue et al. found that supplementing the diet with betaine and choline significantly reduced plasma homocysteine levels in hyperhomocysteinemic rats induced by guanidinoacetic acid. Furthermore, this study was supported by another study in which betaine was supplemented at dose of 4 g/day in healthy humans for 6 weeks, and it was found that betaine lowered the plasma homocysteine level (Setoue et al., 2008; McRae, 2013). Furthermore, in some trials, the betaine molecule also maintains the function of the testicles and sperm cells, especially the concentrations of spermatic ATP. For instance, a study reported that an alteration in the choline dehydrogenase enzyme due to the rs12676 genotype is predicted as the cause of male infertility. However, when dietary betaine was administered, the abnormality in choline dehydrogenase activity was restored. Betaine also improved sperm ATP levels and cell progressive motility (Johnson et al., 2012). Studies also show that betaine has potential as a treatment for MASH patients. Both a notable histological improvement and a significant improvement in aminotransferase levels were observed in MASH patients after a year of betaine treatment (Abdelmalek et al., 2001). Additionally, infection with HCV is a main contributor to CLD. Conventional treatment, pegylated interferon (pegIFN) in combination with ribavirin, has previously shown that the expression of HCV proteins impairs JAK-STAT signaling by inhibiting STAT1 methylation. Since unmethylated STAT1 can bind to and be inactivated by protein inhibitor of activated STAT1 (PIAS1), treating cells with S-adenosyl-L-methionine (AdoMet) and betaine may restore STAT1 methylation and improve IFN signaling (Duong et al., 2006). All these studies indicate that betaine has a wide clinical scope for the management of multiple disorders, including cardiovascular and metabolic diseases.

## 6 Safety and toxicity

Acute toxicity studies are summarized in Supplementary Table S4. In rats, betaine, cetyl betaine, lauryl betaine, and C12–C14 alkyldimethyl betaines had oral LD_50_ values of 11.1 g/kg, 1.62 g/kg, 0.071 g/kg, and 3 mL/kg, respectively. The intravenous LD_50_ of betaine in mice was found to be 0.83 g/kg. In an intraperitoneal study on rats, the LD_50_ values were 0.15 g/kg for cetyl betaine and 0.053 g/kg for lauryl betaine (Burnett et al., 2018; Ridout et al., 1991).

The maximum dose of betaine (5%) evaluated in repeated-dose experiments did not show any effects in rats. In a 91-day oral trial, rats receiving up to 0.35 g/kg/day of cetyl betaine showed no obvious harmful effects. The lowest observed effect level (LOEL) and the no observable adverse effect level (NOAEL) for coco-betaine in a 90-day oral test in rats were 500 mg/kg/day and 250 mg/kg/day, respectively. Oral rat studies testing C12 to C14 alkyl dimethyl betaines up to 300 and 1,000 mg/kg/day, respectively, showed systemic NOAELs of 50 and 100 mg/kg bw/day. Due to increased salivation, elevated urea, and non-neoplastic histopathologic alterations in the kidney and bladder, the systemic LOAEL (low observable adverse effect level) for both of these studies was 150 and 300 mg/kg bw/day, respectively (Burnett et al., 2018; Ridout et al., 1991). In another study involving a 104-week feeding trial with rats, betaine showed no signs of carcinogenicity up to a 5% threshold (Burnett et al., 2018; Ridout et al., 1991).

In multiple studies, betaine has been shown to have anti-irritant effects on the skin. In rabbit studies, coco-betaine at 16% was non-irritating, and in a human study, it was less irritating than sodium lauryl sulfate. Lauryl betaine 0.1% caused no irritation, and reactions occurred for 1% and 10%. Rabbit tests with C12 to C14 alkyldimethyl betaines provided inconclusive results. In some tests, irritation was observed at 30%, but not in others. Betaine (up to 50%), coco-betaine (up to 5%), lauryl betaine (0.1%), and C12 to C14 alkyldimethyl betaines (up to 100%) were not sensitizing in skin tests. However, case reports of allergy to coco-betaine have been reported (Nicander et al., 2003a; GREEN, 2013; Nicander et al., 2003b; Rantanen et al., 2003).

## 7 Translational challenges and limitations

Despite the substantial body of evidence supporting the beneficial effects of betaine in treating multiple disorders, there are still several challenges that hinder its integration into translational practice. A major limitation of the current evidence base is its reliance on pre-clinical studies. Most mechanistic studies have primarily used *in vitro* and *in vivo* models, such as *ApoE*-deficient mice, db/db mice, animals fed a high-fat diet, and disease conditions induced by chemicals. However, despite the consistent demonstration of anti-inflammatory, antioxidant, anti-apoptotic, osmoprotective, and methyl-donor-related effects, it remains uncertain whether these findings can be translated to human disease. Although numerous clinical studies have evaluated betaine, the clinical evidence is less consistent than the evidence from corresponding pre-clinical studies. This may be due to a lack of large randomized clinical trials, along with differences in metabolism, disease progression, and pharmacokinetics between species.

An additional challenge is the incomplete understanding of the molecular mechanisms of betaine. While there is substantial evidence to support its role in one-carbon metabolism, homocysteine metabolism, osmoprotection, and epigenetic regulation, the relative contribution of these pathways to disease prevention remains mechanistically underexplored. For example, the mechanisms underlying the reported interactions between betaine, gut microbiota, GLP-1 signaling, and FGF21 regulation require further detailed studies in both pre-clinical and clinical subjects. The challenge extends beyond underexplored mechanisms to include the inadequately defined therapeutic dose range. For example, betaine doses in clinical studies vary significantly, ranging from 20 mg to 20 g per day. This variation complicates the comparison of studies. Furthermore, analysis of registered clinical trials reveals important barriers to translation. As several betaine-related clinical studies have been completed, many trials have been withdrawn, terminated, or have an unclear publication status. Additionally, several completed studies have not been published in peer-reviewed journals, creating gaps in the published literature. These gaps contribute to reporting bias and hinder a thorough assessment of betaine’s therapeutic potential. Taken together, these limitations highlight the need for well-designed studies with meaningful endpoints. Overcoming these challenges is essential for translating the promising biological effects of betaine observed in experimental studies into evidence-based therapeutic applications.

## 8 Conclusion and future perspectives

**FIGURE 9:**
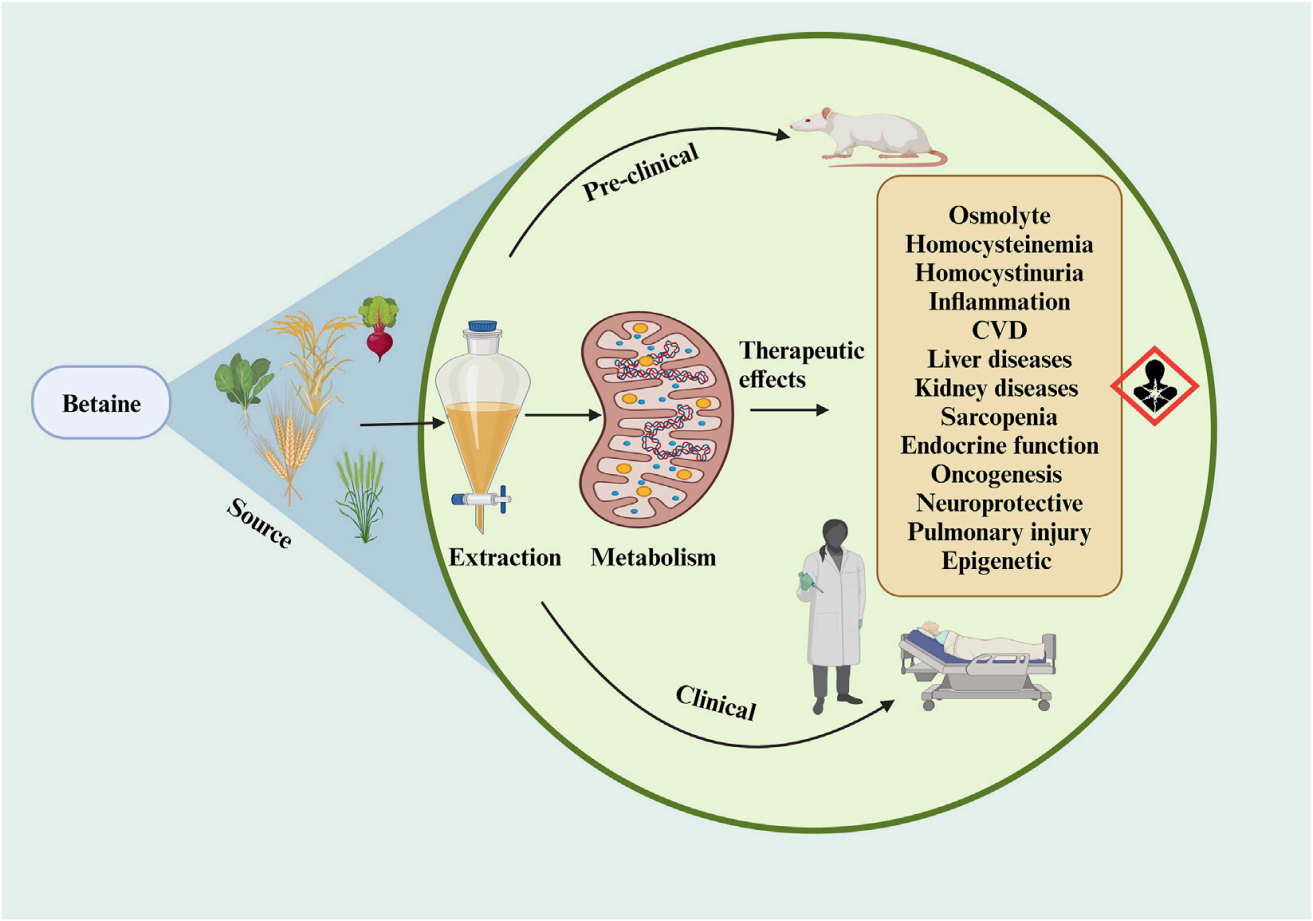
Overview of betaine sources, metabolism, and therapeutic relevance. Betaine is obtained from dietary sources such as wheat, spinach, beetroot, and other plant-derived foods. Following extraction and intake, betaine undergoes cellular metabolism, where it functions as an important osmolyte and methyl donor. Pre-clinical (illustrated by animal models) and clinical studies indicate multiple therapeutic effects associated with betaine. These include modulation of hyper homocysteinemia and hyper homocystinuria, anti-inflammatory actions, and potential benefits in cardiovascular disease (CVD). Additional reported effects involve liver and kidney diseases, sarcopenia, endocrine regulation, oncogenesis, neuroprotection, pulmonary injury, and epigenetic mechanisms. Together, the diagram summarizes the translational pathway of betaine research from dietary sources and metabolic processing to pre-clinical and clinical applications.

Betaine is a glycine derivative that is consumed as part of the diet, with therapeutic properties (Figure 9). It has properties such as being an antioxidant, anti-inflammatory, osmolyte, and methyl donor. Betaine has also been studied for its potential benefits against various metabolic, renal, and cardiac disorders, among others. The outcomes of studies have shown betaine’s protective effect against multiple disease conditions. However, there is a lack of available detailed mechanistic studies on betaine, highlighting the need to explore its mechanism of action against disease-specific models. Furthermore, betaine should be tested in clinical studies to confirm its safety and efficacy. Special attention should also be given to advanced delivery strategies, including nano-formulation, to increase target specificity against disease conditions. Studies indicate that betaine could be a preferable option for treating various disease conditions without major toxicity concerns.
