# Supplementary material for: Betaine as a regulator of metabolism, epigenetics, and cellular osmoprotection: therapeutic implications in cardiometabolic and renal diseases
Source: Front Pharmacol. 2026 Jul 31;17:1883688. doi: 10.3389/fphar.2026.1883688 (PMC13473388; doi:10.3389/fphar.2026.1883688)
Supplement: Supplementary file 1 [file Table1.docx]

Supplementary Material

# Supplementary Table 1: Completed clinical trials with betaine

| S.No. | NCT Number | Condition | Intervention | No. of Participants | Dose | Outcome Measures | Phases | Date | Result | Locations |
| --- | --- | --- | --- | --- | --- | --- | --- | --- | --- | --- |
| 1 | NCT02758015 | Healthy | Betaine hydrochloride | 9 | Betaine hydrochloride (1500, 300 and 4500 mg) p.o. | Heidelberg capsule monitors gastric pH after a meal for 15 minutes. | NA | 2016-2018 | 4500 mg of betaine hydrochloride to have the greatest effect. | University of California, San Francisco |
| 2 | NCT01838941 | Peroxisome Biogenesis Disorders | Betaine | 12 | Betaine given orally 6 g/day in children less than 30 kg, (2 g at mealtime) in 3 divided doses 12 g/day in children more than 30 kg, (3 g at mealtime and bedtime) in 4 divided doses | Measuring the plasma C26/C22 for 6-month. | Phase3 | 2013-2015 | NA | Montreal children hospital, Canada |
| 3 | NCT02404337 | Homocystinuria | Betaine | 12 | Betaine 100 mg and 200 mg | The study compares plasma levels of total homocysteine after oral treatment with betaine at 10 weeks. | Phase 2 | 2015-2018 | NA | Assistance Publique Hôpitaux de Paris, Paris, 75019, France |
| 4 | NCT05790070 | Blood Flow Restriction  Training | Dietary supplement betaine | 20 | 3 g/ twice daily | The study measures genes like FAK, HIF-1, BGT-1, and MHC related to skeletal muscle adaptation | NA | 2021-2021 | NA | Baylor University, Waco, Texas, 76798, United States |
| 5 | NCT00586911 | Non-alcoholic Steatohepatitis | Betaine or Cystadane | 26 | Betaine 20 mg a day for 1 year. | Changes in degree of steatosis, necro inflammatory activity and fibrosis in liver biopsy for 1 year | Phase 2 | 2003-2006 | NA | University of Florida at Gainesville, Gainesville, Florida, 32610, United States Mayo Clinic, Rochester, Minnesota, 55905, United States |
| 6 | NCT01950039 | Obesity Dysglycemia | Betaine | 28 | Betaine administered orally for 12 weeks | Measured glucose disposal and endogenous glucose production at different insulin infusion rates for 12 weeks. | Phase 2 | 2014-2018 | 16.5-times rise in plasma dimethylglycine, but only moderate increases in serine and methionine levels (1.3 and 1.5 times, respectively) in the betaine group as compared to the placebo group. | Joslin Diabetes Center and Brigham and Womens Hospital, Boston, Massachusetts, 02215, United States |
| 7 | NCT01213719 | Healthy Subjects | Dietary supplement contains creatine, betaine, dextrose and creatine + betaine | 40 | Creatine: 20 g/d for 10 days; betaine: 2 g/d for 10 days; dextrose: (placebo), 20 g/d for 10 days; creatine plus betaine; creatine (20 g/d) + betaine (2 g/d) for 10 days | Strength after 10 days | NA | 2010-2011 | NA | University of Sao Paulo, Sao Paulo, 05508-030, Brazil |
| 8 | NCT01237353 | Pharmacodynamics | Dietary supplement: betaine hydrochloride; Drug: Rabeprazole | 6 | Betaine hydrochloride 1500 mg p.o. on day 5 and Rabeprazole p.o. daily x 5 days | Gastric pH levels monitored after administering betaine hydrochloride with a Heidelberg pH capsule, after 2 hours of betaine dose | NA | 2011-2011 | NA | University of California San Francisco, San Francisco, California, 94143, United States |
| 9 | NCT00283387 | Hyperoxaluria | Betaine, Placebo | 16 | Oral betaine 12 grams/day in subjects less than 10 years of age, and 20 g/day in subjects 10 years of age and in more than 10, two equally doses, for 2 months | Urinary oxilate excretion was measured after 2 months. | Phase 2 | 2007-2011 | betaine dosages can successfully treat paediatric patients with homocystinuria resistant to VB6 | Mayo Clinic Hyperoxaluria Centre, Rochester, Minnesota, 55905, United States |
| 10 | NCT02118142 | Insulin Sensitivity | Dietary supplement: 1. Betaine, 2. Dextrose | 23 | Encapsulated betaine/Dextrose dose (6 g) | Plasma concentrations of FGF21, choline, betaine, glucose, insulin, and adiponectin are measured | NA | 2014-2014 | NA | UNC Nutrition Research Institute, Kannapolis, North Carolina, 28081, United States |
| 11 | NCT02122211 | Male Infertility | Betaine supplement | 6 | 11 capsules of powdered betaine (0.5g) administered twice per day (6 in the morning, 5 in the evening) | Changes in sperm motility, count, mitochondrial function, ultrastructure, choline dehydrogenase concentration, and betaine concentration over a 75-day treatment period is evaluated | Phase 1 | 2014-2016 | Check the safety and effects of betaine treatment on men with CHDH rs12676 minor alleles and sperm mitochondrial ATP concentrations | UNC Nutrition Research Institute, Kannapolis, North Carolina, 28081, United States |
| 12 | NCT01749982 | Arsenic Metabolites Measured in Urine | Choline bitartrate (700 mg), Betaine (1000 mg) and Choline bitartrate (700 mg) + Betaine (1000 mg) orally daily | 60 | Choline bitartrate, Betaine and Choline bitartrate + Betaine | Changes in urinary % monomethyl arsenic, inorganic arsenic, and dimethyl arsenic | Phase 4 | 2012-2014 | NA | Columbia University Arsenic Research Project, Dhaka, Bangladesh |
| 13 | NCT02403804 | Healthy | Dietary supplement: Phosphatidylcholine(588mg), betaine (412 mg) q.a.m. and betaine (1000 mg BID) | 8 | Week 1: Phosphatidylcholine 3600 mg q.a.m. and 2700 mg q.p.m Week 2: Betaine anhydrous 588 mg q.a.m and 412 mg q.p.m Week 3: Betaine anhydrous 1000 mg BID | Change in serum choline, 4 hours post supplement. - baseline | NA | 2015-2015 | NA | University of Colorado Anschutz Medical Campus, Aurora, Colorado, 80045, United States |
| 14 | NCT00348933 | Angelman Syndrome | Drug: Betaine, creatine, metafolin and vitamin B12 | 90 | Betaine 100-200 mg per/kg/day orally. Creatine 200 mg per/kg/day orally. Metafolin 0.5 mg per/kg/orally. Vitamin B12 1 mg/day orally | The study analyses the development of speech, communication, cognitive, and daily living skills using Bayley Scales of Infant. | NA | 2006-2010 | NA | Rady Children's Hospital San Diego, San Diego, California, 92123, United States Children's Hospital Boston, Boston, Massachusetts, United States Greenwood Genetics Center, Greenwood, South Carolina, United States Baylor College of Medicine, Houston, Texas, United States |
| 15 | NCT00102843 | Cardiovascular Diseases | Supplementation with folic acid and betaine | 40 | NA | Plasma homocysteine concentrations and Vascular function in a fasting state measured by flow-mediated vasodilation | NA | 2002-2003 | NA | Wageningen Centre for Food Sciences, Wageningen, 6703 HD, Netherlands |
| 16 | NCT01398046 | Healthy | Dasatinib, Dasatinib + Rabeprazole, and betaine hydrochloride | 10 | Dasatinib (100 mg), Dasatinib (100 mg) + Rabeprazole (20 mg) and Betaine hydrochloride (1500 mg) p.o. twice daily (days 1-3); (day 4) Dasatinib (100 mg) plus Rabeprazole (20 mg) and betaine hydrochloride (1500 mg) p.o. x1 | On day 4, the study compares Dasatinib AUC values from 0 to 22 hours with an active comparator. | Phase 1 | 2011-2014 | NA | Clinical Research Center, UCSF, San Francisco, California, 94143, United States |
| 17 | NCT00226356 | Unipolar Depression | Supplements of L-methionine, betaine and folate | 20 | NA | Depressive symptoms | Phase 4 | 2004-2008 | NA | Cambridge Health Alliance, Cambridge, Massachusetts, 02139, United States |
| 18 | NCT01534858 | Burns | Prontosan wound gel X | 51 | Topically, a thin layer of betaine containing hydrogel is placed to the whole grafted area immediately after transplant and with each time the dressing is changed. | The timeline for graft neo-epithelisation is 29 days. | NA | 2012-2015 | NA | Berufsgenossenschaftliche Unfallklinik Ludwigshafen, Ludwigshafen, Baden-Württemberg, 67071, Germany, Berufsgenossenschaftliches Unfallklinikum Bergmannsheil, Bochum, 44789, Germany, Klinikum St. Georg, Leipzig, 44129, Germany |
| 19 | NCT00126347 | Cardiovascular Diseases | Supplementation with betaine, serine, and folic acid | 40 | NA | Study assessed the plasma homocysteine concentrations before and after an oral methionine load. | NA | 2002-2003 | NA | Wageningen Centre for Food Sciences, Wageningen, Netherlands |
| 20 | NCT05635929 | Mucositis | Composition comprising olive oil, xylitol, and betaine | 65 | Topical formulation for use as a mouthwash in the form of a gel and diluted in water | Oral Mucositis Symptom scale and medical team observations. | NA | 2022-2023 | After first 6 weeks of treatment oral mucositis severity and quality of life in H&N cancer patients is examined | Mucosa Innovations S.L., Madrid, 28023, Spain |
| 21 | NCT05342272 | Xerostomia | Betaine taurine PVP gel | 40 | For 28-day treatment, 1-2 cm of gel to the tongue, gums, and oral mucosa is applied | Speed and duration of the products' effects and their pleasantness of use is measured | Phase 3 | 2017-2018 | NA | ASST Spedali Civili di Brescia, Brescia, BS, 25123, Italy  ASST Spedali Civili di Brescia, Brescia, Italy |
| 22 | NCT00310336 | Hepatitis C | S-adenosyl-L-methionine, betaine, pegylated interferon alpha2b. ribavirin | 30 | NA | Sustained response rate | Phase2, Phase3 | 2006-2009 | NA | University Hospital Basel, Basel, BS, 4031, Switzerland |
| 23 | NCT01048307 | Venous Ulcer Care Chronic Wound Care | Prontosan wound irrigation solution (contains 0.1 percent betaine and 0.1% polyhexanide) | 20 | During dressing change the wound bed is cleansed using Prontosan® wound irrigation solution | Study determined bacterial burden, reducing slough and necrotic tissue and assessing granulation tissue quality and quantity once in a week | NA | 2009-2010 | NA | Calvary hospital center for curative and palliative wound care, Bronx, New York, 10461, United States |
| 24 | NCT01759875 | Healthy | Ritonavir-boosted atazanavir, ritonavir-boosted atazanavir + rabeprazole and betaine hydrochloride | 8 | 20 mg Rabeprazole p.o. twice a day (days 1-3); Ritonavir (100 mg) and betaine hydrochloride (1500 mg) p.o. x 1 p.o. x 1 and 300 mg of atazanavir **o**n **d**ay 4, p.o.x1 | Atazanavir's AUC values from 0-22 hours and compares experimental arms with the active comparator is measured | Phase 1 | 2013-2014 | NA | Clinical Research Center, UCSF, San Francisco, California, 94143, United States |
| 25 | NCT01371357 | Hyperhomocysteinemia | TEST (guanidinoacetic acid + betaine hydrochloride+ vitamin B12 + vitamin B6 + folic acid) | 40 | 2.4 g/day of guanidinoacetic acid + 1.6 g/day of betaine hydrochloride+ 5 µg/day of B12 + 10 mg/day of B6 + 600 µg/day of folic acid | Total plasma homocysteine for 3 months | Phase 3 | 2011-2011 | NA | Center for Health, Exercise and Sport Sciences, Belgrade, 11000, Serbia |
| 26 | NCT04566549 | Skin Problems | Test group Probiotic Shampoo | 21 | Shampoo base with probiotics including cocamidopropyl betaine | The scalp microbiota will be examined using NGS or qPCR analysis | Phase 2 | 2020-2021 | NA | Chia Nan University of Pharmacy and Science, Tainan, 71710, Taiwan |
| 27 | NCT01981343 | Gallbladder | A-F Betafood | 50 | 2 tablets with a meal, X3 daily for 12 weeks | Liver and gallbladder function, includes changes in liver function tests (such as fasting serum AST, ALT, GGT, and hsCRP) during a 12-week period | Phase 2 | 2013-2018 | Betafood® may have a beneficial effect on gallbladder function. | London, Ontario, Canada, N6A 5R8 KGK Synergize Inc. |
| 28 | NCT01052298 | Disease, Pulmonary | Ciprofloxacin betaine | 25 | The initial single dose of 48.75 mg ciprofloxacin betaine, equivalent to 75 mg. | After 28 days of the first treatment measures, electrocardiogram, pulmonary function test, and pulse oximetry. | Phase 1 | 2009-2009 | NA | Mannheim, Baden-Wurttemberg, 68167, Germany |
| 29 | NCT00843466 | Hand Dermatosis | Mild, moisturizing hand cleanser | 46 | Hand Sanitizer containing Cocamidopropyl Betaine | Changes in global disease severity on a scale from 0-4 | NA | 2007-2008 | NA | Wake Forest University Health Sciences Dermatology, Winston-Salem, North Carolina, 27157, United States |
| 30 | NCT00961038 | Pulmonary Disease, Chronic Obstructive | Ciprofloxacin betaine | 19 | Ciprofloxacin betaine (32.5 mg) | Measures ECG, pulmonary function test, and pulse-oximetry within 12 days. | Phase 1 | 2009-2010 | NA | Hannover, Niedersachsen, 30625, Germany |
| 31 | NCT03431597 | Hyperhomocysteinemia | Novel micronutrient | 298 | Drink powder form contains 800 μg folic acid, trimethylglycine (betaine) etc. | The effect of a new micronutrient supplement on the plasma homocysteine levels in the drink powder and control groups is compared for 12 weeks | Phase 2 | 2018-2018 | NA | Keneba Field Station, Banjul, Gambia |
| 32 | NCT01939145 | Wound Infection | Prontosan (containing 0.1%) betaine) | 100 | Negative pressure wound therapy containing 0.1% betaine | Number of operative rooms visits between Prontosan NPWTi and normal saline NPWTi is compared for two weeks | Phase 4 | 2013-2016 | NA | Medstar Georgetown University Hospital Center for Wound Healing, Washington, District of Columbia, United States, 20007 |
| 33 | NCT03611283 | Xerostomia Sjogren's Syndrome | Mouthwash treatment (Aqua, Betaine) | 28 | Mouthwash 60 sec. after meals (3 times/day) for 28 days | Validated visual analogue scale questionnaire for xerostomia | NA | 2016-2017 | NA | NA |

# Supplementary Table 2: Ongoing clinical trials with betaine

| S.No. | NCT Number | Condition | Intervention | No. of Participants | Dose | Outcome Measures | Phases | Date | Result | Locations |
| --- | --- | --- | --- | --- | --- | --- | --- | --- | --- | --- |
| 1 | NCT06042270 | Body temperature changes; body water dehydration; Inflammation | Betaine | 20 | Dietary Supplement: Betaine 3 g/day for 4 weeks | Study examines how BET supplementation affects the heat response of professional fireman during live burn training by using measurements like heart rate, skin temperature, and core temperature. | NA | 2024-2024 | NA | Institute of Sports Science and Medicine Tallahassee, Florida, United States, 32301 |
| 2 | NCT04585295 | Healthy | Betaine | 13 | Betaine 50 mg/kg twice daily for 7 days | Fluid compartment volumes, thermoregulation, heat shock protein 72 expression, skin and core temperatures, thermal feeling, enzyme-linked immunoassays, and subjective self-reported thermal sensation were all analysed for 7-day | NA | 2020-2023 | NA | Institute of Sport Sciences and Medicine at Florida State University, Tallahassee, Florida, 32304, United States |
| 3 | NCT06091917 | Childhood Overweight | Betaine supplementation in infant formula | 50 | infant formula supplemented with betaine (100 µmol/L) for 12 weeks | To evaluate supplement safety, infant growth changes, and potential intestinal microbiome changes induced by increased betaine intake | NA | 2023-2025 | NA | NA |
| 4 | NCT05463484 | Oral Dysbiosis | Test | 100 | Toothpaste including a Saliactive® (contains betaine and xylitol). | The study analyzed changes in baseline microbiota, salivary flow rate, pH, bleeding index, plaque index, and elastase over four months | PHASE2\|PHASE3 | 2021-2023 | NA | Departamento de Estomatología, Facultad de Odontología Granada, Spain, 18071 |
| 5 | NCT03337360 | Male Subfertility | Impryl (Food supplement with betaine) | 1200 | 1 tab. daily for 6 months | Ongoing rate of pregnancy | NA | 2018-2024 | NA | Canisius-Wilhelmina Hospital, Nijmegen, Gelderland, Netherlands\| |
| 6 | NCT06045832 | H. Pylori Infection | MAXPOWER Biological Antibacterial Liquid | 42 | MAXPOWER Biological Antibacterial Liquid mouthwash, contains betaine | Using a saliva test (HPS) the oral H. pylori eradication rate was assessed. | NA | 2023-2023 | NA | Changhai hospital, Shanghai, Shanghai, 200433, China |
| 7 | NCT05720702 | NAFLD | Homocysteine (Hcy) lowering supplements | 20 | Hcy lowering supplements contains (Vitamin B12, Folate, Vitamin B6, and Betaine) daily during 12 weeks period | NASH Diagnose, based on elevated NAFLD activity score and significant fibrosis score, | NA | 2023-2024 | NA | Durham, North Carolina, United States, |
| 8 | NCT05743283 | Wound Heal | SynEx wound cleanser | 100 | SynEx wound cleanser containing betaine | Size, depth, edges, and undermining of wound is measured | NA | 2023-2024 | NA | MedStar Washington Hospital Center Washington, District of Columbia, United States, 20010 |
| 9 | NCT06148662 | Oral Health | Dental foam "Biorepair PERIBIOMA, Dental foam "WATER: DENT" and Dental spray "BUCCOTHERM" | 30 | Rinse their mouth for 60 seconds with dental foam "Bio repair PERIBIOMA" | The minimal pH value will be recorded. | PHASE4 | 2023-2023 | Salivary pH duration and the effects of oral sprays on salivary pH levels is measured | Institute of Dentistry of Sechenov University, Moscow, Russian Federation |
| 10 | NCT06122467 | Acne | Exfoliating cleanser | 36 | NA | Change in the appearance of facial skin acne and Changes in skin clarity is examined | NA | 2023-2023 | NA | Citruslabs, Santa Monica, California, 90404, United States |
| 11 | NCT06018168 | Premenstrual Syndrome | MINDBODYSKIN Supplement containing betaine hydrochloride | 60 | 3 capsules daily with a meal, | Virtual skin grading, blood tests to measure vitamin A levels | NA | 2023-2024 | NA | Citruslabs, Santa Monica, California, 90404, United States |

# Supplementary Table 3: Withdrawn or terminated clinical trial with betaine

| S.No. | NCT Number | Condition | Intervention | No. of Participants | Dose | Outcome Measures | Phases | Date | Result | Locations |
| --- | --- | --- | --- | --- | --- | --- | --- | --- | --- | --- |
| 1 | NCT00882193 | Chronic hepatitis C | Betaine; Drug: Peginterferon alpha 2a; Drug: Ribavirin | 2 | For 48 weeks, administer 20g of betaine daily in two doses. weekly subcutaneous injection of peginterferon alpha 2a 180 mcg/0.5 ml and 200 mg of ribavirin, divided into two doses | For a duration of 72 weeks, the safety and effectiveness of betaine, a drug for chronic hepatitis C genotype 1, has been assessed when combined with standard antiviral therapy. | Early Phase 1 | 2009-2011 | NA | University of Nebraska Medical Center, Omaha, Nebraska, 68198, United States |
| 2 | NCT00571714 | Chronic hepatitis C | Peginterferon alpha-2a, ribavirin and betaine | 0 | For a total of 36 weeks during the first twelve weeks of treatment, a once-weekly subcutaneous injection of 180 mcg of peginterferon alpha-2a, ribavirin 800-1400 mg twice a day, and betaine twice a day is administered. | NA | NA | 2008-2010 | NA | NA |
| 3 | NCT01554644 | Diabetic complications | Prontosan^TM^ Wound Irrigation Solution (PHMB 0.1%, Betaine 0.1%) | 0 | ProntosanTM Wound Irrigation Solution contains (PHMB 0.1%, Betaine 0.1%) | NA | NA | 2012-2012 | NA | University of California San Diego San Diego, California, United States, 92130 |
| 4 | NCT03401749 | Surgical site infection | Theraworx Bath Wipes | 203 | Theraworx Bath Wipes (contain Betaine) | NA | Phase 4 | 2018-2020 | NA | Good Medicine, Oxford, Ohio, 45056, United States |
| 5 | NCT04633044 | Overweight and obesity | Dietary Supplement: Betaine | 50 | 400 mg of betaine daily for 12 weeks | Weight and length will be measured for the study at several intervals, like at birth, 1 month, and 3 months. | Phase1  Phase2 | 2021-2022 | NA | Hospital Sant Joan de Deu, Barcelona, 08950, Spain |
| 6 | NCT03073343 | Non-alcoholic fatty liver disease, non-insulin dependent diabetes | Betaine (trimethyglycine) | 48 | for the first four weeks, 4g of betaine per day (2g p.o. BID); after that, 4g per day, or up to 8g per day for an additional eight weeks | Study measures alanine aminotransferase (ALT) levels in participants for 12-week course of Betaine administration | NA | 2013-2021 | NA | VA Long Beach Healthcare System, Long Beach, California, 90822, United States |
| 7 | NCT03702205 | Supplementation | Betaine supplementation | 80 | For a duration of three weeks, each athlete in the study will be given either 2.5 g or 5 g of betaine daily. | After three weeks of betaine administration, the study examines at changes in anaerobic capacity, fat mass, and fat free mass. | Phase 2 Phase3 | 2018-2021 | NA | Institute of Human Nutrition and Dietetics, Poznan University of Life Sciences, PoznaÅ 60-624, Poland |
| 8 | NCT03720249 | Hyperhomocysteinemia | Supplementation of compound nutrients | 100 | Betaine, zinc, and (6S)-5-methyltetrahydrofolic acid are all combined into an 800 mg tablet along with vitamin B6 and vitamin B12. Orally, for 12 weeks, four pills once daily. | In blood sample homocysteine levels in two groups at baseline, four weeks, and twelve weeks is measured | NA | 2019-2022 | NA | Guangzhou, Guangdong, China, 510080 SunYat-sen University |
| 9 | NCT02837367 | Dyslipidemia | Administration of supplements containing methyl-donors | 600 | Methyl-donors (capsules) with the following contents: 2 g betaine, 800 ug (micrograms) 5-MTHF + 1000 ug (micrograms) = L-5-methyltetrahydrofolate Choline bitartrate (500 mg), vitamin B12, 1 g ALA (alfa-linolenic acid), 700 mg EPA (eicosapentaenoic acid), and 280 mg DHA (docosahexaenoic acid) | Examine Triglycerides (TG), LDL-cholesterol (LDLc), HDL-cholesterol (HDLc) | NA | 2016-2019 | NA | Clinica II Pediatrie Bega, Timisoara, Timis, Romania \|Spitalul Judetean Timisoara; Centrul de Diabet, Timisoara, Timis, Romania |

# Supplementary Table 4: Betaine toxicity studies

| **Oral** | | | |
| --- | --- | --- | --- |
| **S.no.** | **Ingredient and concentration/dose** | **Method** | **Results/conclusions** |
| 1 | Anhydrous betaine, (97%) in water at doses 5, 10, 12.5, 15, 20 g/kg/b.w. | OECD Guideline 401: 5 rat/sex/dosage for acute oral toxicity in (SD) rats | 11.1 g/kg b.w. (LD_50_ calculated) male and female, symptoms include ataxia, prone posture, reduced motor activity. |
| 2 | Cetyl betaine (94.9%) and lauryl betaine (98.9%) in 25% (wt./vol) | 5 male Sprague Dawley rat groups were given either test substance orally by gavage. | Loss of appetite, diarrhea, and lacrimation were the symptoms. Distended gastrointestinal tract and mottled lungs were seen during gross necropsy, and there were no appreciable variations between the two test materials. |
| 3 | Coco-betaine (30%) active ingredient 10% in water at doses 6,670, 8,350, 10,000 mg/kg b.w. | OECD Guideline 401: 10 mice/dosage for acute oral toxicity in CF-1 mice | 2,640 mg/kg b.w., (LD_50_ calculated) for 8,800 mg/kg body weight (b.w.) |
| 4 | betaines, C12-C14-alkyldimethyl (Lauryl + myristyl betaine), at doses 0, 1.6, 2.5, 3.2, 4.0, 5.0, and 8.0 mL/kg b.w. in an aq. solution | OECD Guideline 401: 5 rats/sex per dosage for acute oral toxicity in CFY rats | 3 mL/kg b.w. (LD_50_ estimated) in both men and females with lethargy and diuresis were seen at 3.2 mL/kg, ataxia and coma preceded death and happened between 5 and 27 hours after dosage |
| **Dermal** | | | |
| 1 | Lauryl (98.9%) and cetyl (94.9%) betaine | 5 male (S.D.) rats were split up into groups and administered test material topically on trunks that had been clipped | >16 g/kg and 1.3 g/kg (LD_50_) values for cetyl and lauryl betaine respectively, test sites showed signs of erythema, edema, desquamation, necrosis, and scab formation for both test materials. |
| 2 | Betaine; no detail further | Acute study in mice; no detail further | LD50 = 830 mg/kg b.w. |
| 3 | 5% and 25% (wt./vol) solutions of cetyl betaine and lauryl betaine at (94.9%) (98.9%) respectively in distilled water | 5 male (S.D.) rats were divided into groups and given each test substance i.p. | 150 mg/kg and 53 mg/kg (LD_50_) for cetyl betaine and for lauryl betaine respectively. animals showed signs of lethargy, diarrhoea, lacrimation, and distended abdomen |
| **Repeated dose toxicity studies** | | | |
| 1 | In animal feed, betaine (95%) with dosages of 0%, 1%, 2%, and 5%. | As per OECD Guideline 407 | NOAEL >5,771 mg/kg b.w./d |
| 2 | in animal feed, Betaine (95%) | OECD 408 (S.D.) rats, both male and female, were tested for 90 days oral toxicity using repeated doses: 20 rats of each sex | Due to the high tolerance, mild hematological abnormalities, and hepatic alterations, NOAEL and NOEL could not be determined. |
| 3 | Betaine (99.9%) | OECD Guideline 453 for investigations on Fischer 344 rats (male and female) 10 rats of each sex were used in each dosage in a 52-week investigation, and 25 rats of each sex were used in a 104-week study. | NOEL determined for up to 5%, betaine, showing no carcinogenic sign; lower mean corpuscular hemoglobin and mean corpuscular volume were seen in the 5% dosage group, along with high liver and high kidney weights in both sexes. |
| 4 | Coco-betaine tested at 0, 125, 250, and 500 mg/kg b.w./d with 29% to 33% active material in water. | OECD Guideline 408 for 90-day oral toxicity following repeated doses in both male and female (S.D.) rats (10 rats/sex/dosage). | 250 mg/kg b.w./d, 500 mg/kg b.w./d; NOEL and LOEL respectively |
| **Reproductive and developmental toxicity** | | | |
| 1 | Betaines, C12-C14-alkyldimethyl (lauryl + myristyl betaine) at 0, 50, 150, or 300 mg/kg b.w./d in water | As per OECD Guideline 422 in Wistar rats, both male and female, by oral gavage; 10 animals per sex/dose; males administered for 4 weeks, females for 7 weeks; microscopic evaluation of the paternal reproductive organs | Symptoms like drowsiness, salivation, and irritation in the stomach and bladder are shown in high-dose group of parental animals due to the test material's irritating properties, no adverse effects seen in the low-dose group or their offspring. |
| 2 | betaines, C12-C14-alkyldimethyl (Lauryl + myristyl betaine) at 0, 33, 100, 300, or 1,000 mg/kg b.w./d in water | As per OECD Guideline 422 for oral gavage treatment of male and female Wistar rats for repeated dosage toxicity study in combination with reproductive/developmental toxicity; 10 animals per sex/dose; males administered for 4 weeks, females for 6 weeks; microscopic evaluation of parental reproductive organs | In the 1,000 mg/kg/d dose group all parental animals died within 24 hours of the first dose. The test substance's NOAEL for reproduction was 300 mg/kg/d, and no adverse effects on reproduction were noted at 300 mg/kg/d |
| **Genotoxicity** | | | |
| **In-vitro** | | | |
| 1 | Betaine monohydrate (95%) | human lymphocytes in whole blood cultures with and without metabolic activity for the investigation of chromosome aberration | Not prone to clotting |
| 2 | Betaine monohydrate (97%) Conc. plated range from 8 to 5,000 µg/plate, with and without S9 activation | Salmonella typhimurium strains like TA98, TA100, TA1535, and TA1537 with and without S9 metabolic activation were used in the bacterial reverse mutation test.; | No sign of genotoxicity |
| 3 | Betaines, C12-C14-alkyldimethyl (combination of Lauryl betaine + myristyl betaine up to 5.0 mg/plate | In accordance with OECD Guideline 471. S. typhimurium strains TA97a, TA98, TA100, TA1535, and TA102 with and without metabolic activation for bacterial reverse mutation (Ames) assay | No sign of genotoxicity |
| 4 | Alkyl dimethyl betaine (30.2% active ingredient) with S9, up to 100 µg/m and without S9, up to 75 µg/mL | In accordance with OECD Guideline 476.In vitro mammalian cell gene mutation studies are performed using Chinese hamster ovary (CHO) cells, which contain the HGPRT locus with or without metabolic activation. | No sign of genotoxicity |
| 5 | Alkyl dimethyl betaine (30% active ingredient), with 4-hour exposure and without S9 up t0 200 µg/mL, with 20-hour exposure and without S9 up to 100 µg/mL and up to 150 µg/mL with S9 | According to OECD Guideline 473, mammalian chromosomal aberration in vitro studies in CHO cells with and without metabolic stimulation | No sign of genotoxicity |
| **In vivo** | | | |
| 1 | monohydrate Betaine (98%) in saline at doses 0, 0.5, 1, or 2 g/kg | As per OECD Guideline 474 for mammalian erythrocyte micronucleus test. mice were exposed for 24, 48, or 72 hours and cyclophosphamide, the positive control, was given by gavage to male and female CD-1 mice | dosed up to 2 g/kg in mice bone marrow did not produce micronuclei. |
| **Irritation** | | | |
| **Dermal non-human** | | | |
| 1 | Coco-betaine t (16%) | OECD Guideline 404: in rabbits (albino)occlusive on shaved and abraded skin; for acute cutaneous irritation/corrosion | The mean score for erythema and edema is 0.5/4, and it can be fully reverted within 24 hours. Not irritating |
| 2 | Betaines, C12-C14-alkyldimethyl  (Lauryl betaine + myristyl betaine), Concentration not mentioned | As per the OECD Guideline 404, 3 New Zealand white rabbits, semi occlusive on shaved skin for severe cutaneous irritation/corrosion | Between 30 and 72 hours after the dose, there was a little edema, moderate erythema, and the appearance of dry, rough skin and scales. effects that are completely reversible after 14 days. Considered irritating |
| 3 | Betaines, C12-C14-alkyldimethyl  (Lauryl betaine + myristyl betaine), Concentration not mentioned | As per OECD Guideline 404 in 3 New Zealand white rabbits, semi occlusive on shaved skin, for acute cutaneous irritation/corrosion | Mean erythema score: 1.83/4; mean edema score: 0.83/4; neither completely reversible within 72 hours; not irritating |
| **Dermal human** | | | |
| 1 | Betaine (10%) | Two studies (n=28 and n=21) involving healthy patients evaluated the test material's efficacy in reducing irritation when used in soap. | Betaine-containing soap was found to be less irritating than non-test-material soap |
| 2 | Betaine (5%) in  Various vehicles | 2 occlusive Finn patches were applied to 26 healthy volunteers for two consecutive 24-hour periods; the test site area = 50 mm^2. | Not irritating |
| 3 | With 2% sodium lauryl sulphate (SLS) and betaine (95%) at 3.5% in water | 20 male and twenty female test subjects applied test materials to different arms for four weeks. | Considered an anti-irritating agent, betaine has been shown to reduce the irritating effects of SLS |
| 4 | Coco-betaine and 4 surfactants | In a TEWL investigation, 27 healthy volunteers' dermal irritation was determined using Finn chambers: 24 hr exposure period | Coco-betaine had been found less irritant than SLS |
| 5 | Lauryl betaine (as 0.1% active component) | Acute skin irritation in 19 human participants; exposure for 30 hours; non-occluded | No reactions noted. |
| 6 | Lauryl betaine as an 1% and 10% active component | Acute skin irritation in 7 humans subjects; occluded; 24-hour long exposure | 1 strong, 4 moderate, and 2 mild erythema in the 10% solution, compared to 5 strong, 1 moderate, and 1 mild erythema in the 1% solution |
| **Ocular—Animals** | | | |
| 1 | Betaine monohydrate (95%)  10% (wt./vol) in distilled water | For acute ocular irritation/corrosion in albino rabbits, in accordance with OECD Guideline 405 | Not irritating |
| 2 | Coco-betaine (16%) | For acute ocular irritation/corrosion in albino rabbits, in accordance with OECD Guideline 405 with unwashed eyes | At the day 7; postdosing, the test substance had induced corneal involvement and conjunctival irritation that had not reduced considered as irritating |
| 3 | Coco-betaine (as 30% active  Component) | For acute ocular irritation/corrosion in albino rabbits, in accordance with OECD Guideline 405 with unwashed eyes | At the day 7; postdosing, the test substance had induced corneal and iris involvement and conjunctival irritation that had not reduced considered as irritating |
| 4 | Lauryl betaine (10% vol/vol) in distilled water | For acute ocular irritation/corrosion in albino rabbits, in accordance with OECD Guideline 4053 New Zealand white rabbits with unwashed eyes | Irritating |
| **Mucosal human** | | | |
| 1 | Betaine (4%) in a toothpaste | In 20 individuals, the study examined the effect of betaine on lowering mucosal irritation in toothpastes containing SLS | The oral mucosa was found to be irritated by toothpastes containing SLS and betaine, though toothpastes containing 4% betaine alone did not irritate the mucosa |
| 2 | Betaine (4%) in a toothpaste | 13 participants participated in a study to investigate betaine's ability to reduce "dry mouth" caused by SLS toothpaste. | No adverse effect on the toothpaste containing 4% betaine |
| **Sensitization** | | | |
| **Animals** | | | |
| 1 | monohydrate Betaine (97%) | Groups of ten female Dunkin-Hartley guinea pigs were used in the guinea pig maximisation test, in accordance with OECD Guideline 406 for skin sensitization. | Found not sensitizing |
| 2 | Betaines, C12-C14-alkyldimethyl  (Lauryl betaine + myristyl betaine) at 5%, 10%, 25%, 50% (wt./vol) in ethanol/water | For LLNA in CBA mice,4 animals/dose in accordance with OECD Guideline 429 | After the second administration, erythema was observed |
| 3 | Betaines, C12-C14-alkyldimethyl (combination of Lauryl betaine + myristyl betaine) as 0.1% of 30% active component | Draize test: (all male) six Dunkin-Hartley guinea pigs | Not found sensitizing |
| 4 | Betaines, C12-C14-alkyldimethyl (combination of Lauryl betaine + myristyl betaine) | 20 female Himalayan patched guinea pigs and 10 control animals were used in the Buehler test for skin sensitization in accordance with OECD Guideline 406. | During the first and second weeks of induction animals showed no signs of systemic toxicity or mortalities, but in the third week 12/20 experienced discrete/patchy to moderate confluent erythema. |
| **Human** | | | |
| 1 | Lauryl betaine (as 0.1% active ingredient) | HRIPT in 20 Subjects | After seven days, subjects experienced a strong reaction after induction, followed by a mild reaction. |
